# A Hybrid Human-Neurorobotics Approach to Primary Intersubjectivity via Active Inference

**DOI:** 10.3389/fpsyg.2020.584869

**Published:** 2020-12-01

**Authors:** Hendry F. Chame, Ahmadreza Ahmadi, Jun Tani

**Affiliations:** Cognitive Neurorobotics Research Unit (CNRU), Okinawa Institute of Science and Technology Graduate University (OIST), Okinawa, Japan

**Keywords:** social cognition, interaction theory, neurorobotics, human-robot interaction, free energy principle, developmental psychology, educational technology, cognitive rehabilitation

## Abstract

Interdisciplinary efforts from developmental psychology, phenomenology, and philosophy of mind, have studied the rudiments of social cognition and conceptualized distinct forms of intersubjective communication and interaction at human early life. *Interaction theorists* consider *primary intersubjectivity* a non-mentalist, pre-theoretical, non-conceptual sort of processes that ground a certain level of communication and understanding, and provide support to higher-level cognitive skills. We argue the study of human/neurorobot interaction consists in a unique opportunity to deepen understanding of underlying mechanisms in social cognition through synthetic modeling, while allowing to examine a second person experiential (2PP) access to intersubjectivity in embodied dyadic interaction. Concretely, we propose the study of primary intersubjectivity as a 2PP experience characterized by predictive engagement, where perception, cognition, and action are accounted for an hermeneutic circle in dyadic interaction. From our interpretation of the concept of *active inference* in *free-energy principle* theory, we propose an open-source methodology named *neural robotics library* (NRL) for experimental human/neurorobot interaction, wherein a demonstration program named *virtual Cartesian robot* (VCBot) provides an opportunity to experience the aforementioned embodied interaction to general audiences. Lastly, through a study case, we discuss some ways human-robot primary intersubjectivity can contribute to cognitive science research, such as to the fields of developmental psychology, educational technology, and cognitive rehabilitation.

## 1. Introduction

At present, technology has permeated distinct spheres of human society, which has fundamental implications for the research of cognition. Considering the field of robotics, in order to cope with challenges of our times, it is important to study how the inclusion of robots can transform the economic and social organization of our society (Granulo et al., 2019), and possible ways of dealing with negative consequences of those changes. It is also crucial to explore how human-robot interaction can serve beneficial purposes, such as helping in advancing the state of the art in cognitive science, which is a guideline of our research.

In this evolving context, several research communities with distinct purposes have dedicated themselves to study human-robot interaction in recent years. An aspect of central concern is how to include the robot in interaction research. During interdisciplinary conferences and encounters in our field, this matter is manifested through the question: *who is the subject in the interaction?*. From a methodological level of analysis, we could roughly distinguish three main responses to this question. That is, some studies include human-as-subject and robot-as-input (HS/RI), others include human-as-input and robot-as-subject (HI/RS), and probably very few studies, like the current one, include human-as- and robot-as- subject (HS/RS). Next, we describe briefly these approaches.

In HS/RI studies the internal structure of the artificial partner is considered of secondary importance to the analysis of experimental protocols, continent upon the quality of subjectivity is placed on the human partner side. Thus, the robot is commonly included as a typified black-box system that provides some degree of input standardization, and observations are carefully conducted on the human partner's side. Under this perspective, robots have been considered for assistance therapy to help humans recover from sensory-motor deficits in some neuro-psychological conditions (Gassert and Dietz, 2018). Robots have also contributed to the acquisition of computational thinking skills (Atmatzidou and Demetriadis, 2016), improving executive functions of planning and control (Di Lieto et al., 2020), sensory-motor gaming (Kose-Bagci et al., 2009), and metacognitive and problem solving (Atmatzidou et al., 2018). Interaction has also served to study neural development in *autistic spectrum disorder* (ASD, Robins et al., 2005; Scassellati et al., 2012; Ismail et al., 2019). Among several other studies that could be mentioned.

On the other extreme, HI/RS research is founded on the interest in studying human cognition, under the expectation that plausible theories in cognitive science can be constructed through modeling and carefully implementing cognitive control schemes in robots. Here, the human partner acts by stimulating the robot during interaction. Thus, observations on the human side are considered in terms of evaluating experimental hypotheses on the synthetic prototype. Within this perspective, behavior-centered studies have focused on robot tasks that could be directly mapped to analogous research with human infants and children (Cangelosi and Schlesinger, 2015). Since the knowledge available in developmental cognitive robotics is insufficient (Asada et al., 2009), implementations are proposed from the designers' limited understanding of cognitive functions (e.g., to study foundations of communication, Kuniyoshi et al., 2004). The research conducted in our lab has taken inspiration from brain sciences (Tani, 2016), for the proposal of composable continuous state space neuro-dynamic structures (e.g., Tani, 2003; Murata et al., 2013; Ahmadi and Tani, 2019) to study cognition.

In this work, we argue human/neurorobot interaction constitutes a unique methodological opportunity to deepen understanding of underlying mechanisms in social cognition through synthetic modeling. Unlike other accounts, our research treats both the human and the robot as agents involved in an engaged continuous flow of interaction (i.e., a HS/RS perspective), and studies the concept of *primary intersubjectivity* in developmental psychology, through on-line dyadic direct interaction. For this, we provide an integrative model of agency and primary intersubjectivity, as it functions between the human and robot, based upon the interaction of top-down deliberative processes and bottom-up perceptual processes. From our interpretation of the concept of *active inference* in *free-energy principle* theory (Friston et al., 2013; Allen and Friston, 2018), we investigate interaction as a process of coupling, in which neither agent resort to reading the “inner” or “mental” goals, beliefs, and desires of the other. Instead, understanding the other, in the form of emerging coordination, is achieved through direct perception of embodied activity. We propose an open-source methodology named *neural robotics library* (NRL) for experimental research, and through a study case, we discuss some ways human-robot primary intersubjectivity can contribute to cognitive science research, such as to the fields of developmental psychology, educational technology, and cognitive rehabilitation.

## 2. Hybrid Primary Intersubjectivity

*Intersubjectivity* is a concept that carries deep philosophical roots. It is fundamental to Hursserl's foundations on phenomenology (Husserl, 2013), and received important contributions from Heidegger (with the notion of *being-in-the-world*, Heidegger et al., 1962), Merleau-Ponty (through the study of perception and embodiment, Merleau-Ponty, 1996), and Habermas (in the *theory of communicative action*, Habermas and McCarthy, 1984), among several other sources. Dealing with the philosophical complexities of the concept would certainly surpass our current scope. Thus, our focus is rather placed on the much more circumscribed sphere of developmental psychology, and concerns the capacity of understanding others' intentions, actions, feelings, behaviors, or thoughts; through interaction. Below, we establish five important scope delimitations of our work, which concern: the experiential level of analysis, the theoretical perspective, the sort of interaction, the perspective on knowledge representation and prediction, and the nature of the interaction partners.

A conceptual distinction in social cognition research has been established to define the sort of access a person uses in understanding another person. According to Fuchs (2013), from an experiential level of analysis, the access possibilities to oneself and others conform the triad: first (1PP), second (2PP), and third (3PP) person perspective. Hence, subjective experiences are accessible from 1PP, co-experiences or intersubjective experiences (reciprocal interaction, forms of mutual relatedness) are accessible from 2PP, and one-way, vicarious, or remote observations are accessible from 3PP. An intense debate has been established concerning the experiential level of analysis from which studying intersubjectivity. As a first scope delimitation, our research focuses on 2PP, which has been less explored in the literature, probably due to the methodological challenges it imposes.

Concerning the theoretical scope delimitation, several authors (e.g., Gallagher, 2001; Fuchs, 2013; Newen, 2018) have pointed out that studies of social cognition have traditionally explained how individuals understand and interrelate with each other from the perspective of theory of mind. Notably, under the approaches of *theory theory* (TT) and *simulation theory* (ST). In essence, from a 3PP experiential level of analysis, TT theorists have investigated intersubjective relations as specialized cognitive abilities for explaining and predicting behavior, based on the employment of folk psychological theories about how behavior is informed by mental states (e.g., Premack and Woodruff, 1978; Leslie, 1987; Wellman, 1992; Gopnik and Schulz, 2004). ST theorists have studied intersubjectivity from a 1PP experiential level of analysis, as how mental experience becomes an internal model for understating the other's mind, so thoughts or feelings of the other person are simulated as the subject would be in that situation (e.g., Davies and Stone, 1995; Gallese and Goldman, 1998; Goldman, 2006; Rizzolatti and Fogassi, 2014).

Developmental theorists have challenged theory of mind accounts of social cognition, and described stages in the development of intersubjectivity (Gallagher, 2008; Spaulding, 2012). Thus, *primary intersubjectivity*, originally described by Trevarthen (1979), consists in a voluntary interpersonal communication process characterized by intentionality and adaptation, which is founded in innate, embodied, pre-theoretical, non-conceptual fundamental capacities for self-expression and understanding others (e.g., facial gesticulation; proprioceptive sensation, automatic attunement, detection of intentional behavior, eyes motion tracking, noticing emotions in gestural intonation and expression, among others). Thus, it implies the immediate experience of sharing subjective states, in the sense that babies and mothers are biologically endorsed with the capability to coordinate their actions with the other. This ability facilitates cognitive and emotional learning through social interaction. *Secondary intersubjectivity* is theorized to be constituted later, transcending the face-to-face sort of interactions to a context of shared attention, mediated by communication about objects and events in the environment (Trevarthen and Hubley, 1978). Conforming to Rochat and Passos-Ferreira (2009), the stage of *tertiary intersubjectivity* is characterized by processes of negotiation with others about the values of objects, from shared and self representations.

The study of social interaction, as characterized by primary intersubjectivity, is very interesting for us. As such, from our approach in neurorobotics (which is discussed later in the article), we aim at joining interdisciplinary efforts by developmental psychologists, phenomenologist, and philosophers of mind, which have constituted, in the last decades, a diverse field of research that investigate four central features of cognition. Thus, according to Newen et al. (2018), *4E cognition* is considered to be *embodied, embedded, extended*, and *enactive*. Theorist in 4E cognition research cognitive phenomena as dependent on the body characteristics (on its physiology, biology, and morphology), on the particular structure of the environment (e.g., natural, technological, social), and on the active embodied interaction of the agent with the environment.

When considering the study of 4E properties of social cognition, the critical movement against theory of mind approaches was very much influenced by the proposal of *interaction theory* [IT (Gallagher, 2001), also named *embodied social cognition*; (Gallagher, 2008)]. For IT, experiencing the feelings and intentions of another person is mostly accounted for by a 2PP access to immediate perception of embodied interaction with others, which constitutes a simpler, non-mentalistic, on-line capacity. Hence, mind-reading skills (as studied in TT and ST) consist in specialized forms of intellectual activity less regularly used when basic embodied processes fail to account for a given situation. Moreover, such abilities (e.g., perspective-taking) are believed not to be matured enough at early infancy, where the individual has mostly access to body sensations (proprioception, vision, somato-sensation), and is capable of basic motor skills (e.g., following others' eyes, imitating facial expressions, exerting rudimentary motor control, among others). Although IT is not an uniform theoretical field, according to Newen et al. (2018) theorist share the two following ideas: (a) understanding others does not involve observing others on a regular basis, but interacting with them, and (b) the experiential access in understanding through interaction is immediate or direct perception. Hence, as a second scope delimitation, our research adopts the IT perspective for the study of primary intersubjectivity.

Concerning the third scope delimitation, when analysing differences amongst 4E cognition theorists in the study of intersubjectivity, it is fundamental to precise how the interaction situation is conceived and investigated. In this sense, for enactivist theorists (e.g., De Jaegher and Di Paolo, 2007), social interaction is characterized by coupling, which maintains an identity in the relational domain, and by individual autonomy. An example would be walking in the opposite direction in a narrow corridor, where individuals are attempting to stop interacting but the interaction self-sustains notwithstanding their will. Alternatively, conforming to Reddy (2010), it is not the structure of the situation that determines a 2PP level access experience in the interaction, but the fact of mutual acknowledgment, emotional involvement, or engagement (e.g., sharing a smile, attraction, interest, surprise).

Our research is concerned with engaged interactions as described by Reddy. A distinguishable aspect of our work is the interest in direct interaction experiences characterized by intention and purpose. That is, along with possessing means for adaptation or fitness to other's actions, individuals are also capable of employing volitional resources to express their intention. Consequently, dynamic control is shared within the dyad. We believe that this sort of exchange is possible when both agents are capable of, among several skills, processing feedback and formulating proactive expectations on how the situation would look like while enacting in the dyad (Tani, 2016).

The fourth scope delimitation involves disagreement amongst 4E cognition theorists on the importance of knowledge representation and prediction. Conforming to Schlicht (2018), some theorists have radically departed in a non-representational approach and studied emergent interaction from dynamic system theory, whereas moderate theorists have retained mental representation in theorizing social cognition. Examples of the former are studies of minimalistic interaction (Auvray et al., 2009; Froese and Ziemke, 2009; Lenay and Stewart, 2012; Froese et al., 2014). Concerning moderate views, according to Gallagher and Allen (2018), some interpretations of the general framework of the predictive model are consistent with methodological individualism (e.g., Hohwy, 2013), whereas others are consistent with autopoietic enactivist theories (e.g., Kirchhoff, 2018a,b). Notably, *free energy principle* (FEP) theory (Friston et al., 2013).

This work investigates primary intersubjectivity from the perspective of FEP theory, within the scope of 4E cognition and IT. We selected an interpretation of FEP theory which allows us to study the capacity of understanding others without the need of resorting to third person knowledge representation, but based on direct perception of an error signal in 2PP interaction. In this sense, in agreement with Allen and Friston (2018), we study FEP theory as a synthetic account to explain the constitutive coupling of the brain to the body and the environment. Thus, internal representation and prediction, in the generative sense, are considered to emerge from the organismic *autopoietic*^1^ self-organization. Given the assumption of *ergodic*^2^ dynamical interchange between the agent and the environment, we investigate perception, cognition, and action as explained by an enactive hermeneutic circle taking place in dyadic encounters (Gallagher and Allen, 2018). For this, we study interaction as a process where deliberative control and automatic adjustment coexist.

Finally, concerning the fifth delimitation, when deconstructing the section's title semantics, it is important to discuss the meaning of the term *hybrid*. It accounts for the study of social interaction between two partners distinct in nature. In this sense, an important issue investigated in human-robot interaction has been human engagement, where the *uncanny valley effect* (i.e., emotional response in subjects from perceived human resemblance of synthetic objects, Mathur and Reichling, 2016) has been reported. Differently from this line of research, we focus on the inclusion of robots *as they are* to study interaction, and not on how the robot could substitute the human partner. In the next section, we briefly introduce our approach in neurorobotics, by relating it to previous studies in FEP theory, and describing how it can be useful to study primary intersubjectivity within the perspective of 4E cognition and IT.

## 3. Human/Neurorobot Interaction

Neurorobotic agents are inspired in brain science research. It is generally considered that understanding brain functions requires the integration of knowledge at multiple levels of abstraction (Hawkins and Blakeslee, 2007; Ishii et al., 2011; Freeman, 2014). Thus, synthetic models can be proposed to study distinct aspects of the brain, such as synaptic molecular protein synthesis, how neuromechanical signals are transmitted, how spiking activity in a single neuron unfolds, local cell assembly circuits, and the whole brain network. We are interested in the study of relational and organizational aspects of cognition by taking a synthetic approach. Particularly, we employ recurrent neural networks (RNN), which are highly adaptable nonlinear dynamical systems able to deal with both temporal and spatial information structures.

Several architectures have been investigated in our lab [e.g., *continuous time recurrent neural network* CTRNN (Beer, 1995), and *multiple timescale recurrent neural network* MTRNN (Yamashita and Tani, 2008)]. More recently, a variational framework named *predictive-coding-inspired variational recurrent neural network* (PV-RNN, Ahmadi and Tani, 2019) was proposed. The PV-RNN framework is selected as a case study in this work for two main reasons: (a) to our knowledge it is a unique architecture inspired by foundations of FEP theory, which can perform learning of continuous spatio-temporal patterns, as required for neurorobots, and (b) it is mathematically formulated in relation to the variational inference literature, which characterizes it as a relevant framework for several fields of cognitive science.

Previous research has studied social cognition from FEP theory. Thus, several works (e.g., Hohwy and Palmer, 2014; Lawson et al., 2014; Van de Cruys et al., 2014) have attempted to explain social behavior in ASD through the predictive model account. Commonly, a theory of mind stance has been adopted. This has been also the case for the research of intersubjectivity and communication (e.g., Friston K. and Frith, 2015). In general, studying social cognition is tremendously challenging from the methodological point of view. Regularly, researchers have resorted to off-line computer simulations (e.g., Friston K. J. and Frith, 2015), or to on-line indirect interaction mediated by virtual systems, as a means to exert control over extraneous experimental variables (e.g., the technique *hyperscanning*, Babiloni and Astolfi, 2014).

A peculiarity that emerges in dyadic direct interaction is that actions depend on both partners' interventions. Moreover, several sensory and motor organs are simultaneously involved and not only voluntary control, but spontaneous or covered reactions are produced and regulated by the central and peripheral nervous systems. Noticing this has been particularly important for the study of neural development in ADS (Torres et al., 2013).

**Figure 1:**
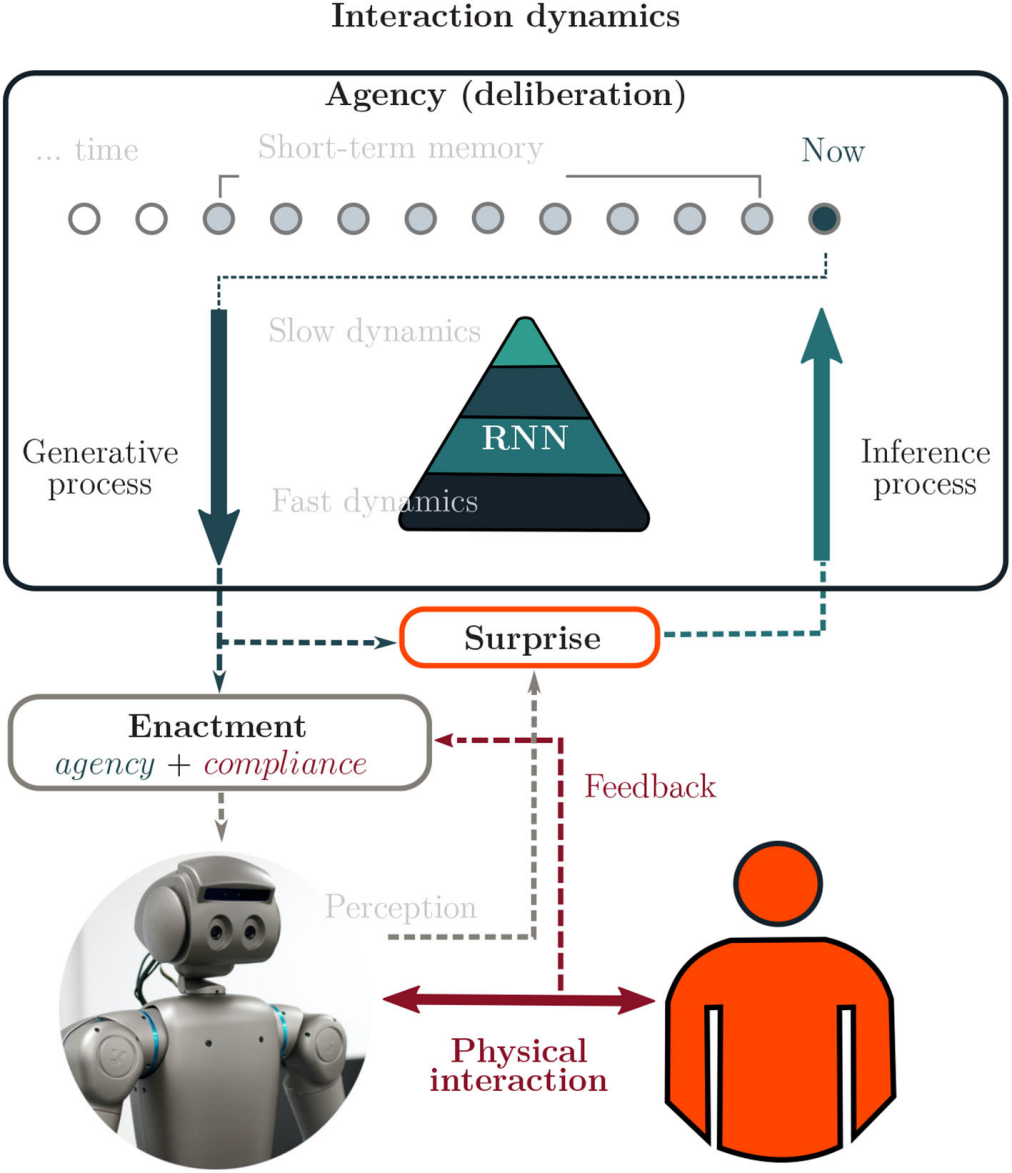
Control is shared in the interaction based on the individuals' capacities of enacting agency and compliance. The capacity of agency is studied through the optimization of free energy (*surprise*) in short-term memory of direct perception. Hence, from the robot's unique past, an intended future is reinterpreted in the hierarchical RNN for deliberation control and projected in the perceptual space. The capacity of motor compliance is modulated as a proportional integral (PI) control scheme for automatic adjustment of the body to the actions induced by the partner.

The representation proposed in Figure 1 for describing human/neurorobot interaction dynamics, takes into account the previous considerations, and the theoretical assumptions adopted in our work. The neurorobot is provided with the capacities of agency (or deliberation) and compliance (reactiveness, adjustment) in relation to the human partner's actions. In agreement with FEP theory (Friston et al., 2011), the robot's deliberative control is studied as a variational optimization process that involves a hierarchical dynamical representation, in which a top-down information flow (developed by a generative process) characterizes the agency of purposeful actions (or intention to behave) in the interaction context, and a bottom-up information flow (an inference process) accounts for their consequences; so conflicts possibly appearing between these two processes are attempted to be reduced through minimizing free energy as a statistical quantity.

Compliance is a fundamental capacity for social interaction which is based on the awareness of others' intentions and knowing what those intentions refer to. This capacity has been studied from a developmental perspective at its early emergence (Reddy et al., 2013; Reddy, 2018). When contextualizing our work in the IT literature, we hold the assumption that automatic processes are available to the person, so the body reacts by adjusting to some extent to the partner's actions. Here, we do not study nor evaluate this assumption, this is an aspect that remains for a further examination. Thereby, we focus on understanding deliberation in the dyad from our interpretation of FEP principle theory, taking automatic adjustment as granted.

Our previous research (Chame and Tani, 2020) has shown in on-line human-robot interaction a possible way in which compliance, as a cognitive (volitional) dynamic process, can relate to reactive motor adjustment. Hence, *cognitive compliance* was studied as a dynamical integrative optimization process that characterizes how deliberation is influenced by sensory stimulation induced by the partner's actions in the dyad. Thus, it was shown how an agent with strong belief tends to act egocentrically to the environment without changing its internal state or intention, whereas an agent with weak belief tends to act adaptively to the environment by easily changing its internal state or intention, while the capacity of motor adjustment was kept constant.

We consider that the perspective adopted here for studying primary intersubjectivity is consistent with the characterization provided by Trevarthen (1979), who views this construct as a deliberative-compliant process. Therefore, we propose to investigate enactivist social cognition in human-robot interaction as a relevant methodological approach for several fields of human science research, which is discussed in section 5. We are aware of the difficulties involved in presenting concepts originated within distinct research fields into a coherent interdisciplinary theoretical formulation. For this reason, we find it pertinent to dedicate the remainder of the current section to introduce such fundamental concepts with minimal mathematical formulation, under the modality of a questions and answers panel discussion.

### 3.1. What Is Free Energy?

Free energy principle theory was initially proposed as a unifying framework in brain science (Friston, 2010). More recently, the theory has been extended and related to the fields of theoretical biology, statistical thermodynamics, and information theory (Friston et al., 2013). A core assumption in FEP theory is that living organisms are driven by the tendency to resist the second law of thermodynamics, so to maintain their internal structure or dynamics in a constantly changing environment.

Conforming to Allen and Friston (2018), from this fundamental drive for existence, biological system are characterized by the following properties: *ergodicity* (an organism occupies or revisits some characteristic states more than others over time in order to live), *Markov blanket* (a mathematical description of the boundaries between the organism and the environment, such description is undertaken at multiple levels of analysis), *active inference* (perception and action are locked in a circular causality relation), and *autopoiesis* (emergent and self-organized maintenance of organismic dynamic structure while interacting with the environment).

FEP theory consists in a predictive account of the mind. It considers that an agent proactively anticipates sensation from empirical priors (in a generative sense), and minimizes free energy as a measure (an upper bound) of *surprise*. As a consequence, the internal state of the agent is maintained within characteristic or habituated regions. Thus, less/more sensory surprise means lower/higher free energy, with more/less likelihood of internal dynamics unfolding in a given region.

### 3.2. How Is Free Energy Minimized?

Generally, free energy is considered to be minimized in two fundamental ways: through *predictive coding*, for vicarious perception, and through *active inference*, for goal directed action (Friston et al., 2011). In predictive coding, surprise is minimized in a bottom-up pathway, so the internal state is modified to generate more consistent predictions with respect to sensory evidence. This principle has been explored in theory of mind studies of social cognition (e.g., Kilner et al., 2007; Koster-Hale and Saxe, 2013; Van de Cruys et al., 2014). Differently, in active inference sensory predictions are attempted to be fulfilled in a top-down pathway, by taking purposeful actions in the environment (Friston et al., 2010).

Regardless of the way surprise is minimized, in FEP theory it is assumed that sensation prediction is accompanied with an estimate of precision. Hence, surprise is considered to be minimized in relation to (divided by) precision, which means that free energy is minimized more when associated with a high precision estimate (i.e., a strong belief). A practical implication of the previous statement is that an agent with strong belief tends to act egocentrically to the environment without changing its internal state or intention, whereas an agent with weak belief tends to act adaptively to the environment, by easily changing its internal state or intention.

This work focuses on the study of enactivist social cognition based on active inference. As pointed out by Friston et al. (2011), in active inference no distinction is established in terms of sensory or motor representations, since motor control signals are considered to be directly generated by proprioceptive predictions, so the individual perceives relevant action affordances in the interaction context. This is going to be discussed in more detail when considering the matter of perception as a direct experience. In the Appendix sections, active inference is described within the scope of our case study, which includes the PV-RNN framework. The minimization of free energy is equivalent to the maximization of the sensory evidence lower bound (ELBO), which is discussed in the next question.

### 3.3. What Is the Evidence Lower Bound?

The ELBO is a quantity introduced in the variational Bayesian (VB) optimization literature. In FEP theory, this quantity corresponds to the subtraction of two terms, namely the *accuracy* term, representing the prediction error, or surprise; and the *complexity* term, representing the complexity of the internal representation. Minimizing free energy is equivalent to maximizing the ELBO. Mathematically, accuracy is the expected logarithm likelihood of the model with respect to the approximated posterior distributions, and complexity is the Kullback-Leibler divergence (KL divergence) between the approximated posterior and the prior hidden distributions. Therefore, by definition *ELBO* ≤ 0 is an upper limit for the logarithm of the marginal likelihood in the anticipation of the sensory state by the generative process (see the mathematical details in the Appendix B section). For human-robot interaction experiments it is perhaps more intuitive to relate surprise to the negative evidence lower bound (N-ELBO), since increases in N-ELBO correlate with situations where mismatch increases between the anticipation and actual sensation.

### 3.4. Is Perception a Direct Experience?

In the study of perception-action, two schools have contrasted, namely, the *contructivist* and the *ecological* theory of perception. These theories have developed over several years. Here, only their basic characterization is presented. Thus, constructivist theorists are very much influenced by the Helmholtz's notion of *unconscious conclusion* (von Helmholtz, 2013). It is generally assumed that stimulation reaching the sensory apparatus is not sufficient for perception, so intermediate processes (e.g., memory, perceptive schemes, previous experience) intervene between sensation and perception, which characterizes an inferential and indirect process. Contrarily, the ecological school, under the influence of Gibson's studies of visual perception (Gibson, 2014), considers that information available in the ambient suffices, since what is perceived are changes over time and space (an information flow). Accordingly, individuals perceive *affordances* (i.e., functional utilities with respect to themselves and their action capabilities) of objects or the environment. Consequently, perception is studied under the ecological school as a direct or immediate process.

Constructivist theories of perception have been associated with cognitivism, when attributing a predominant role to the brain in the top-down information processing of sensory data (e.g., Gregory, 1974), which largely neglects the richness of information available at the sensory level. However, although neurophysiological, neuropsychological, and psychophysical scientific evidence has supported the coexistence of constructivist and ecological perceptive processes in the brain (Norman, 2002), according to de Wit and Withagen (2019), ecological psychologists have been criticized for ignoring the brain in their theoretical formulations.

More recently, Linson et al. (2018) have argued that when replacing traditional inferential explanations, based on the notion of passive input, with the notion of active input, ecological theorists still maintain an input-output account of perception. According to the authors, active inference is characterized in FEP theory as direct engagement (in the thermodynamic sense) between the agent's sensory system and the environment. Thus, sensation is considered to be anticipated from a generative process, which does not constitute an inferential process in the input-output sense. As pointed out by Bruineberg and Rietveld (2019), perception and action from anticipatory dynamics and free-energy minimization ensure the agent with the capability of maintaining adaptive sensibility to relevant affordances in a given context. This would not be about reconstructing the structural hidden causes of the environment.

This debate has been certainly inspiring for artificial intelligence and robotics research. It could be relevant bringing up to discussion influential works by Brooks in behavioral robotics (e.g., Brooks, 1990, 1991). The field was also influenced by Braitenberg (1986). In behavior robotic agents, sensory input has been directly mapped to motor output. This idea would arguably conform to principles of the ecological theory of perception (Tani, 2016). Although interesting behavior can result from minimal control schemes, this sort of robot is only capable of reacting to the environment. Lacking of intention, they behave in stereotyped ways and exhibit low capacity of generalization, even to slightly different situations.

In the approach adopted in our research, robots are capable of active inference, so they perceive action affordances directly as a vector flow in the perceptual space (i.e., no distinction is established in terms of sensory or motor representations). Hence, such neural robots try to fulfill their intention in the interaction by directly performing afforded actions making sense in a given context.

## 4. Case Study: Interacting With VCBot

This section presents the methodological details of the case study. It starts by discussing the relevance of the interaction scenario for the research of primary intersubjectivity, and introducing the proposed tools. Next, the case study is presented in four stages, concerning the modeling and training of the neurorobotic prototype, the report on the interaction experiment, and the analysis of data. Finally, the section discusses how the case study supports the theoretical formulation of primary intersubjectivity.

### 4.1. The Tool Proposed

Differently from the mother-infant interaction study conducted by Trevarthen (1979), which consists in distant face-to-face interaction from visual and auditory feedback, we decided to explore engaged direct interaction between the human and the robot (i.e., both are in physical contact). This is mostly due to the fact that prototyping interoceptive sensory feedback requires of less control of extraneous variables (e.g., the scene disposition, illumination, ambient noise, etc.) for conducting experiments. Furthermore, models based on exteroceptive data tend to be considerably more expensive in terms of computation resources, which would negatively impact real-time performance.

Another important simplification of our studied scenario is that, unlike with infants, we focused on proposing a robotic prototype with a reasonably limited behavior repertory and an exclusive attentional focus in motion generation. In Trevarthen's study it was observed that infants eventually attenuated the production of dyadic goal-directed gestures and did not visibly respond to the mother for a while, by getting distracted with something else or exploring their own body. These alternatives were not provided in the robot prototype.

The case study was also inspired in our previous research (Chame and Tani, 2020), which has shown that both the human and the robot are able to process feedback from each other, so congruent and complementary behavior can emerge in the interaction. An interesting aspect shown in the study is that the robot's behavior did not correspond to an input-output mapping scheme where human intentionality is simulated, but to the development of an artificially embodied form of intentional behavior control.

From the previous considerations, as it is detailed in Appendix A, we propose a methodological resource named *Neural Robotics Library* (NRL)^3^ for designing neural agents. The library can be used to prototype agents (both physical or virtual robots), capable of behavior control for human-robot interaction. During experiments, the subjective state of the agent can be recorded and become a valuable resource to investigate on-line social cognition. This information can be combined with measures on the human side, and allow the study of HS/RS dynamics.

**Figure 2:**
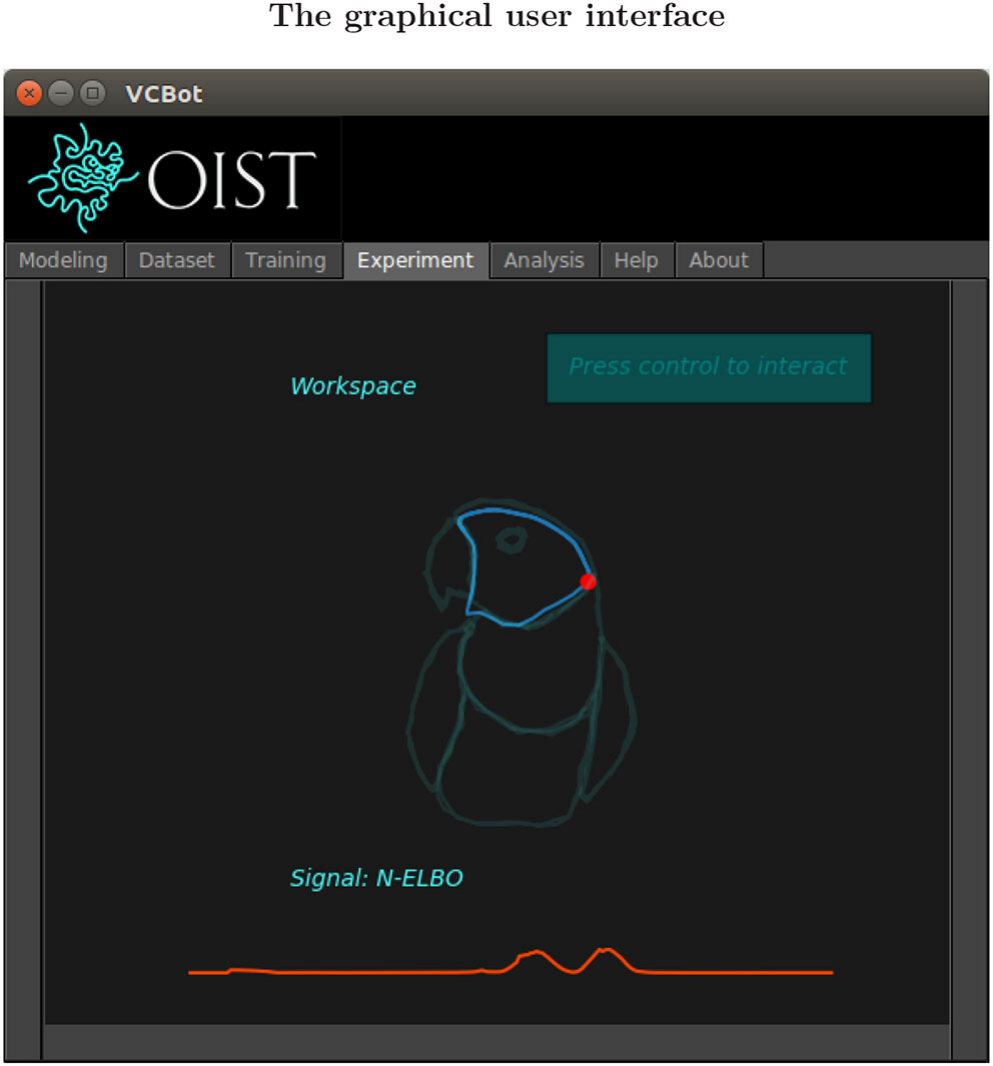
VCBot in experiment mode ready for interaction. The training dataset (in watermark color for reference) includes behavior primitives globally shaping a macaw cub. The end-effector is represented by the red circle. Recent behavior is shown in light blue (the robot is reproducing the shape of the head).

The virtual Cartesian robot, shortly named VCBot, is a program relying on NRL which is designed for interaction with an artificial neural agent through the computer mouse^4^. Compared to real robots experiments, VCBot is a conceptually different platform proposed with the hope of illustrating our theoretical formulation, without counting on robotic hardware. That is, in VCBot the human cannot receive proprioceptive feedback from the robot intention through the mouse, but only observe the trajectory of the end-effector. Concretely, we propose as a case study an interaction task that consists in drawing the body parts of a macaw cub. The graphical user interface (GUI) is conceived following a notebook layout (see Figure 2). It includes in dedicated tabs the four methodological steps implemented in NRL (see Appendix A, Figure 1), which is discussed below.

### 4.2. Modeling

Agent modeling starts by the selection of the network features. The PV-RNN parameters shape important qualities such as compliance in the deliberation style of the agent. Unfortunately, the selection of parameters cannot be done analytically, so the method of trial-and-error should be employed. Table 1 provides a qualitative description of the parameters' role and some hints on their selection. An important aspect to consider is that computational complexity is conditioned to the number of intermediate layers and the amount of neurons within layers. Therefore, it is recommended to start by profiling a reduced structure and gradually increasing its complexity until it is able to handle the task. This iterative process is represented in Appendix A, Figure 1 as the cyclic flow labeled *improvements*. For reference, the models included for demonstration in VCBot comprise two layers: the Low layer is composed of 40 d units, 4 z units, and the time constant set to 2; whereas the High layer is composed of 10 d units, 1 z unit, and the time constant set to 10.

**Table 1:** PV-RNN parameters selection.

**Parameter**	**Description**
z units	Represent the stochastic latent state in the prior and posterior distributions (see Appendix B, Equations 4, 8). In PV-RNN these units encode a Gaussian distribution parameterized by a mean (or expectation) μ and a standard deviation σ, so *z* = μ+σ*ϵ with ϵ sampled from N(0,1).
d units	Represent the deterministic latent state (see Appendix B, Equation 3). As a rule of thumb they are set ten times more numerous than z units.
Regulation *w*	Is a meta-parameter which influences the learning of the posterior and the prior distributions (see Appendix B, Equation 14). In general, the higher the parameter is set, the more similar the hidden prior and posterior distributions would be, so the internal representation would be less sensitive to stochasticity during interaction (when deliberating, the agent would comply less to the partner's intentions). On the other hand, if *w* is set too low, the agent's generative process (based on the prior distribution) would be poor, so deliberation will tend to be erratic.
Timescale constant	The timescale conditions the temporal dynamics of the layers. The constant should be selected increasing proportionally between adjacent layers from the lowest to the highest, so low layers present faster dynamics than higher layers. For example, assuming a configuration of three layers, in case ι^middle^ = 5ι^low^ (see Appendix B, Equation 2), then ι^high^ = 5ι^middle^.

### 4.3. Training

The objective of constituting training sets is to capture fundamental behavior on the robot side for studying during interaction. Thus, depending on the theoretical aspect under consideration, such basic skills could be assumed to be either innate to the agent, or acquired through developmental sensory-motor processes (e.g., through motor babbling, imitation).

A convenient method used for registering behavior primitives is kinesthetic demonstration, where the human directly moves the robot body to show the behaviors. For the case study, a two-dimension motion primitive set was constituted, inspired by the body shape of a macaw cub (perhaps on the species *ara ararauna*). A total number of seven primitives were included, registered at 100 ms sampling period, during 72 time steps, conforming limit cycles in a clockwise sense. These trajectories represented distinct anatomic regions of the bird (i.e., the left eye, the head, the beak, the neck, the right wing, the belly, and the left wing). Only individual primitives were included in the dataset, so the robot did not learn how to relate one primitive to another. Hence, an important aspect to observe is whether possible relations between previous knowledge emerge during interaction.

**Figure 3:**
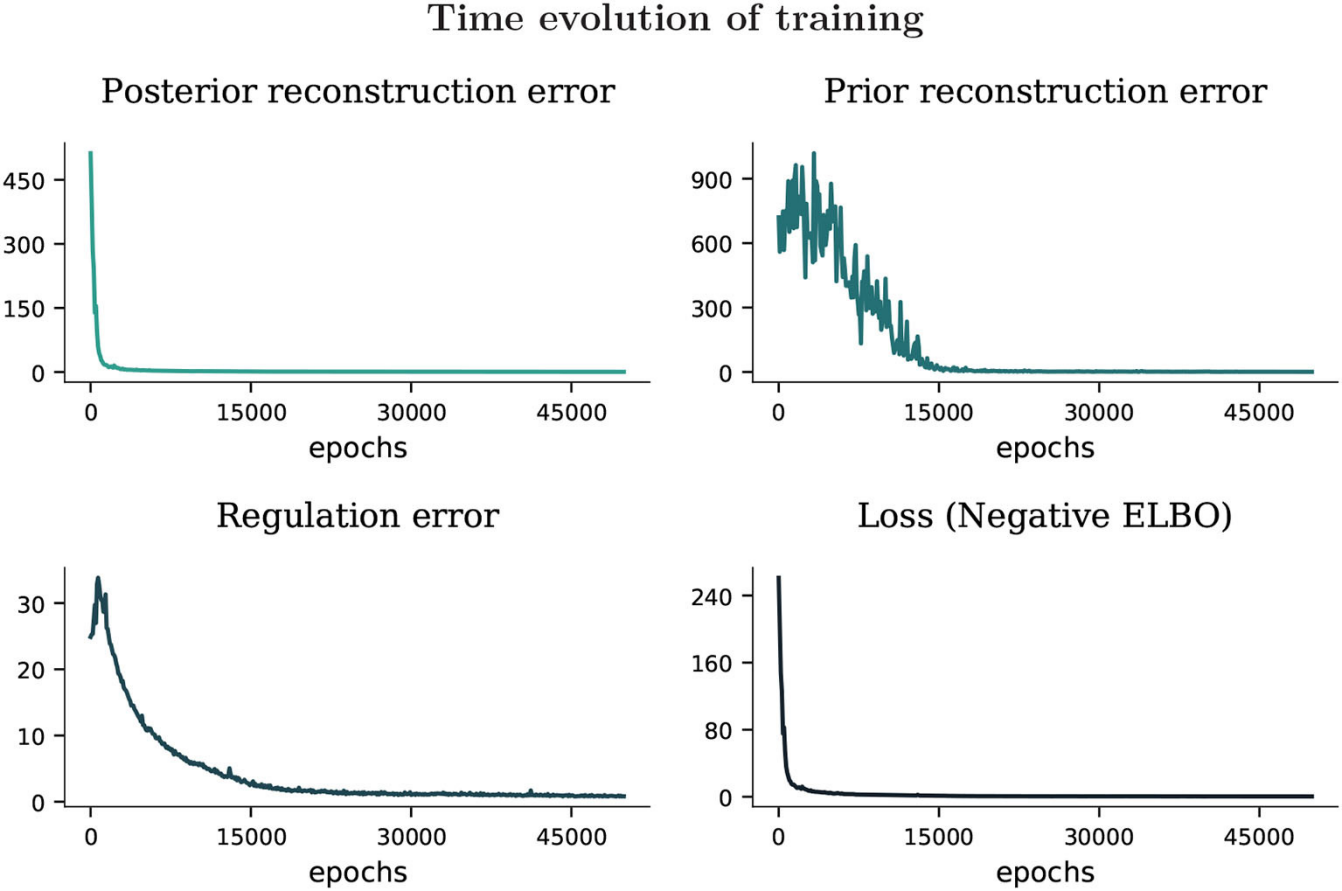
Training was optimized during 50,000 epochs. The parameter selected for the Adam method were α = 0.001, β_1_ = 0.9, β_2_ = 0.999. The posterior and prior reconstruction errors are calculated by taking the softmax transformation on training data along every degree of freedom of the robot, and computing the sum of the Kullbach-Leiber divergence between the training reference and the output generated by the network from the posterior and the prior distribution, respectively.

The model was trained during 50,000 epochs (see Figure 3), following the Adam method for stochastic optimization (Kingma and Ba, 2014). As noticed on the top row, the network optimized faster the reconstruction error (the accuracy component of the ELBO, or the expected logarithm likelihood of the model with respect to the approximated posterior distributions, see Appendix B, Equation 14) related to the posterior distribution, and gradually improved the results for the prior distribution, which can be noticed in the way the signal corresponding to the regulation error decreases (at the bottom-left, it represents the complexity component of the ELBO, that is the Kullback-Leibler divergence between the approximated posterior and the prior hidden distributions). This means a gradual increase of complexity in the model, since the prior and the posterior distributions are becoming more similar. Thus, as seen in the plot on the bottom-right, the negative ELBO is minimized over the training epochs.

**Figure 4:**
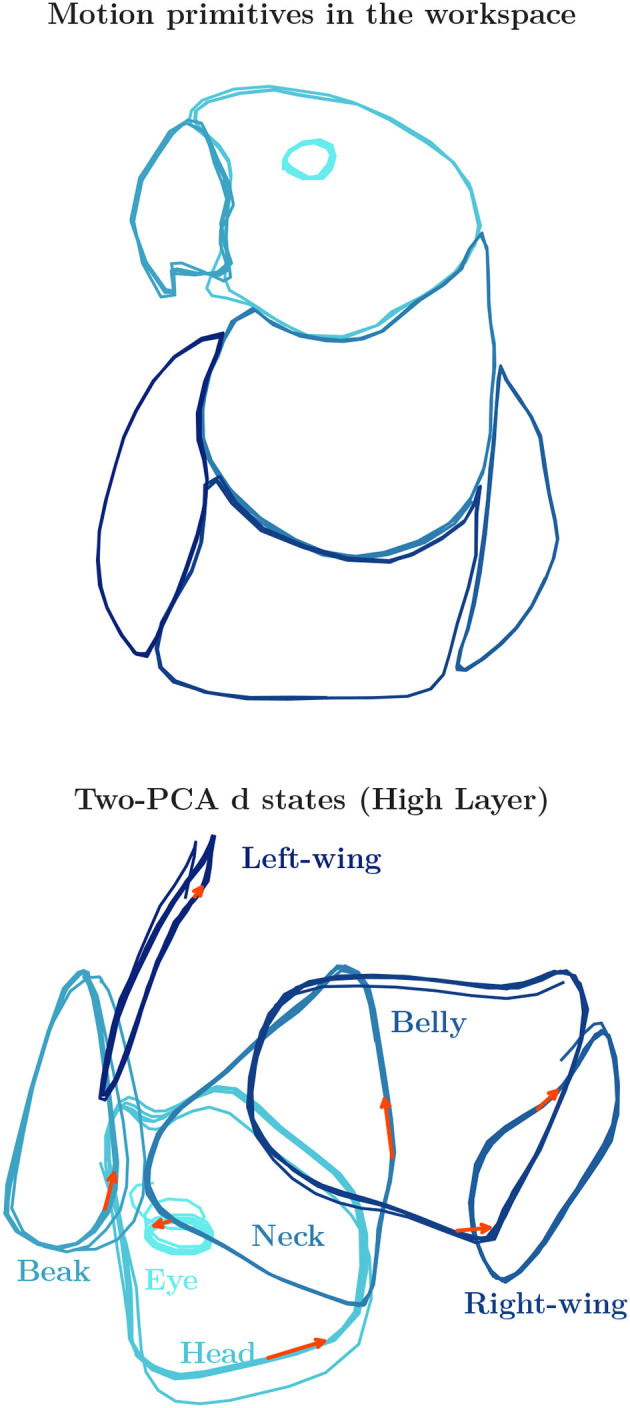
**(Top)** The seven behavior primitives representing globally the body shape of a macaw cub were successfully learned by the prior generation process. **(Bottom)** The resulting internal representation self-organized by the network form training. It is important noticing the connectivity between regions, and the sense of information flow in the attractors developed, which is indicated by the orange arrows.

After training, the primitives were generated from the prior distribution to evaluate the quality of behavior achieved. As seen at the top of Figure 4, the generation process could reproduce in overall the body shape of the macaw cub, as compared with the training set trajectory shown in watermark color in Figure 2. For observing the internal representation self-organized by the network, two principal component analysis (PCA) from the activity of d units at the High layer (slowest dynamics) were plotted at the bottom of the figure. An important aspect to be noticed is the connectivity between regions in the internal representation. Hence, some primitives are represented inter-connected whereas others are not. The absence of connectivity should imply higher difficulty in switching between those attractor regions, this is going to be discussed in more details when analyzing the interaction results. It is also important noticing that although there may be overlap in the regions, the flow of activity could be in the opposite sense. Since all the primitives were captured clockwise, their internal representation also preserved those spatial-temporal relations.

### 4.4. Experiment

The goal of the human-robot interaction experiment was to observe mutual interaction and influence between the partners. For this, the human was instructed to reproduce with the robot the body part of the macaw cub. In the experiment it is not important that all primitives are covered, but how mutual interaction in both directions could develop. Thus, the robot was set to start generating one of the primitives, and the human was instructed to try for each primitive by turn to cover the behavior repertory as much as possible, within a given time. The human was also instructed to proceed at will, so no predetermined order was recommended in trying to accomplish the primitives. After the experiment, the human was requested to verbally report on the primitives attempted with the robot.

A simple proportional controller was designed to endorse the robot with the capacity of enacting deliberation and adjustment (motor compliance) in relation to the human's actions (see Figure 1). Thus, the position of the robot's end-effector emerging from the interaction was determined from the linear interpolation of the human and the robot desired actions, such that *position* = γ*human*−(1−γ)*robot*. For the study case γ = 0.9, which means the human exerted much more influence over the position of the end-effector than the robot did. We set this parameter in this way by considering an analogy to adult-infant interaction situations where the adult's motor system is much more developed and capable than infant motricity. Thus, it would be close to a situation where the mother grabs the infants hand and gently moves it to obtain a response from the infant. In order to avoid brusque changes in the motion trajectory, the position rate of change was saturated by a constant factor.

**Figure 5:**
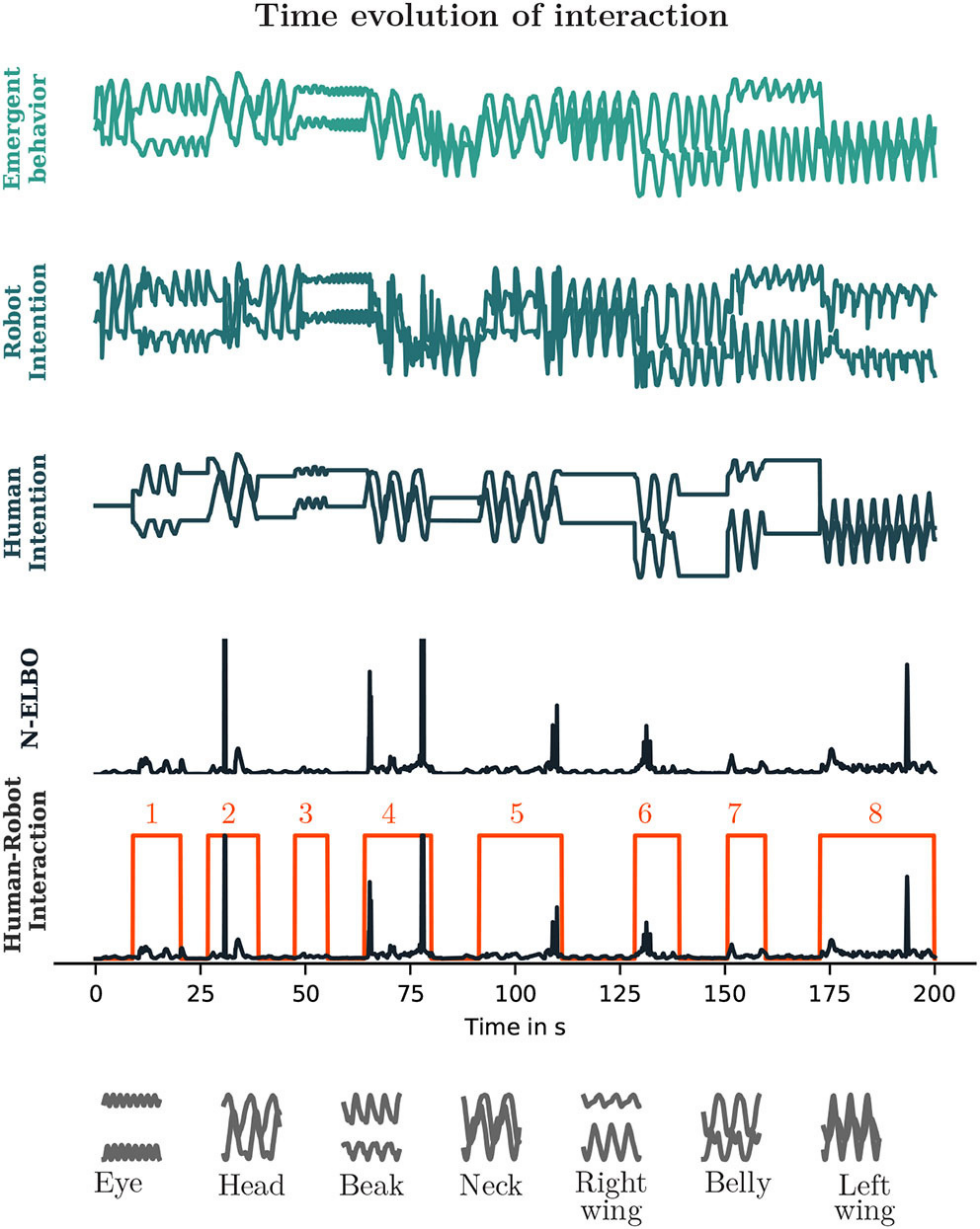
In the plots the axes and labels are not shown for clarity. The vertical component of the signals correspond for the top three plots to the width and the depth coordinates of the robot's end-effector. The N-ELBO is shown in the two lower plots. For the plot Human-Robot Intention, a scaled binary signal representing the control key pressing event by the human is also shown. Temporal contiguous human interventions were grouped and numbered. The horizontal component corresponds for all cases to the time dimension.

In the experiment, the human interacted with VCBot during approximately 3 min and 20 s. Data was captured during 2,000 time steps, at every 100 ms. The human desired actions were registered as the coordinates of the mouse cursor. In VCBot the human chooses when to enable interaction by pressing and holding the control key from the computer keyboard while moving the mouse. Therefore, both the position of the mouse cursor and the key press event were recorded. The robot desired actions were generated by the neural network. The negative ELBO was also recorded as a measure for real-time minimization of free energy. Several repetitions of the experiment were performed, Figure 5 shows the time evolution of data captured for the most interesting trial. A total of eight human intervention events were produced during the experiment. Table 2 presents the human self-reported intention on the events. These results are analyzed next.

**Table 2:** Human intention self-report.

**E**	**Description**
1	The robot was doing Head, the human intended to do Beak, so the robot could switch accordingly.
2	The robot was doing Beak, the human tried to do Head, which the robot accomplished.
3	The robot was doing Head, the human successfully induced a change to Eye.
4	The robot was doing Eye, the human tried Neck for a while, however the robot switched to Left-wing.
5	The robot was doing Left-wing, the human tried again Neck, so the robot could follow up this time.
6	The robot was doing Neck, the human induced a switch to Belly.
7	The robot was doing Belly, the human made it change to Right-wing.
8	The robot was doing Right-wing, the human tried in vain switching to Left-wing.

### 4.5. Analysis

A quantitative measure of intentionality congruence is proposed based on an automatic regression observer. The objective is classifying the robot and the human intention from the observer's evaluation, which receives as input the intended behavior signal, buffered in a limited temporal window, and outputs the attribution of the signal to a behavior category (i.e., a represented body part of the macaw cub). This problem is analogous to some extent to the comparison of time series for speech recognition (e.g., Sakoe and Chiba, 1978). Thus, two conditions are assumed in the comparison: (a) patterns are time-sampled with a common and constant sampling period, and (b) there is no a priori knowledge about which parts of the patterns contain important information.

The automatic regression observer corresponded to a feed-forward model with the following layered structure: 10 input units (2 degrees of freedom ×5 time steps buffer window), 150 units in the first hidden layer, 100 units in the second hidden layer, 7 output units (representing the macaw cub body parts categories). The hyperbolic tangent activation function was selected for the hidden layers, and the sigmoid activation was selected for the output layer.

For constituting the training set, the PV-RNN model generated during 200 time steps each behavior primitive from the prior distribution. Since the initial position was the same for all generations (the center of the workspace), the first 20 generation steps were discarded, in order to ensure the effector position entered the limit cycle attractor of each primitive, consequently, the training sequences had 180 time steps.

The test set was constituted with data captured from the human. The subject was instructed to manipulate the mouse to generate in VCBot the primitives, provided a visual guide on the dataset (data was plotted in watermark color in the workspace). The confusion matrix is presented in Table 3. As noticed, classifications were reasonably accurate for the test set.

**Table 3:** Testing set confusion matrix.

	**Eye**	**Head**	**Beak**	**Neck**	**Rwing**	**Belly**	**Lwing**
Eye	1.000	0.000	0.000	0.000	0.000	0.000	0.000
Head	0.000	1.000	0.000	0.000	0.000	0.000	0.000
Beak	0.000	0.000	1.000	0.000	0.000	0.000	0.000
Neck	0.000	0.000	0.000	0.976	0.000	0.000	0.024
Rwing	0.000	0.000	0.000	0.000	1.000	0.000	0.000
Belly	0.000	0.000	0.000	0.000	0.000	1.000	0.000
Lwing	0.000	0.000	0.000	0.000	0.000	0.006	0.994

**Figure 6:**
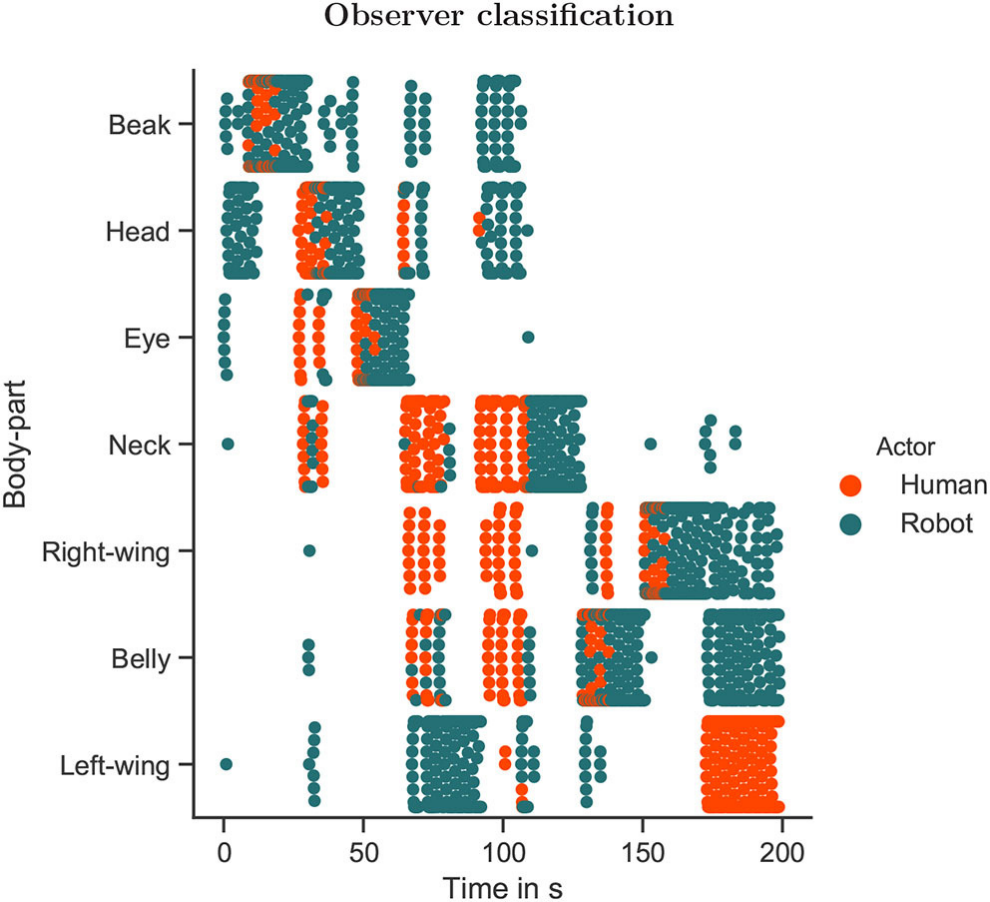
The scatter plot presents the intended action from the human and the robot actors, as evaluated by the observer. The human intervened a total of eight times to influence the robot behavior, which is also illustrated in Figure 5 and self-reported in Table 2.

Figure 6 presents the automatic regression observer's performance for the experimental data. A first aspect to be noticed is that classification for the human intention is available only during the events illustrated in Figure 5 and self-reported in Table 2, whereas data is available from the robot throughout the whole experiment (except for the first 5 time steps, given the size of the temporal buffer window). Although the interaction was subject to stochasticity from the human motions and possible incongruence in the human and the robot intention, temporally correlated classifications for the human actions are observed around a main category and adjacent neighbors, which suggests the human likely focused on particular parts of the bird's body during the interaction events.

In order to estimate the probability of intention congruence for an episode *i* within an interaction event *e*, the binomial variable *c*_*t*_∈{0, 1} is defined from the human ψ^human^ and the robot ψ^robot^ intended actions in the time interval [*s*−*t*^*i*^, *t*^*i*^], so that

(1) $$\begin{array}{l} c_{t}=f\left(\psi_{\left[s-t^{i},t^{i}\right]}^{\text{human}},\psi_{\left[s-t^{i},t^{i}\right]}^{\text{robot}}\right), \end{array}$$

where *t*^*i*^ is the last time step of the episode, and

(2) $$\begin{array}{l} f\left(\psi_{\left[s-t,t\right]}^{\text{human}},\psi_{\left[s-t,t\right]}^{\text{robot}}\right)=\left\{ \begin{array}{cc} 1 & \text{if }O\left(\psi_{\left[s-t,t\right]}^{\text{human}}\right)=O\left(\psi_{\left[s-t,t\right]}^{\text{robot}}\right),\\ 0 & \text{otherwise} \end{array}\right. \end{array}$$

given the observer's classification function $O\left(\psi_{\left[s-t,t\right]}^{*}\right)$.

The probability of intentional congruence for the event *e* at time *t* is defined such that

(3) $$\begin{array}{l} P\left(C_{t}^{e}|\psi^{\text{human}},\psi^{\text{robot}}\right)=\frac{1}{Y}\overset{j=t}{\underset{j=Y-t}{\sum }}c_{t}, \end{array}$$

where *Y* acts as a low pass filter to reduce classification errors due to observation noise.

**Figure 7:**
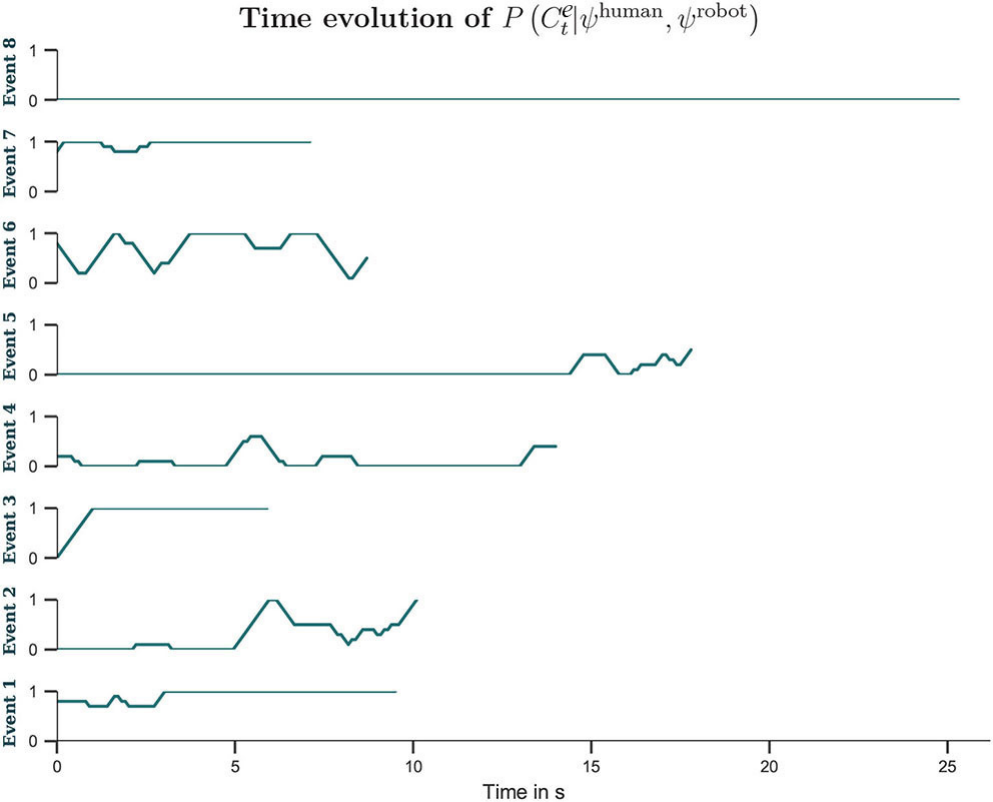
Events unfolded with distinct time length (horizontal axis). The vertical axis shows the probability of intentional congruence (see Equation 3), with *Y* = 10 time steps.

Figure 7 presents the time evolution of the probability of intention congruence, grouped by interaction events. As noticed, for some events congruence was observed earlier in the interaction (e.g., the events 1, 3, 6, 7), so the human was perhaps not too sensitive to feedback from the robot and persisted longer than required to induce intention switching. On the other hand, in the 5th event, although the probability of intention congruence was not very high by the end of the event (it was estimated to be 0.5), once the human ceased to intervene around the time 110 s (see Figure 6), it is clear that the robot switched to the human-self reported intention (see Table 2), so the human was perhaps more sensitive to feedback from the robot in this event. Finally, the events 4 and 8 were mostly characterized by intention incongruence.

### 4.6. What Can Be Learned About Primary Intersubjectivity?

The case study has presented an interaction scenario between a human and a robot, from which one may formulate interesting questions, such as: *what is being shared between agents?, what does each agent “know” about the other?, what do the agents share or coordinate together?, how is this co-regulated in the moment?*, and more generally, *what can be learned about primary intersubjectivity through the case study?*. A good starting point to tackle these issues, is to evoke some important theoretical formulations from the field of developmental psychology. Thus, as described by Rochat and Passos-Ferreira (2009), it is considered that by the age of 2 months basic neonatal imitation (i.e., a mirroring process) gives way to first signs of reciprocation in face-to-face exchanges, which are characteristic of primary intersubjectivity (Trevarthen, 1979). These embodied interactive practices would constitute the primary access through which we understand others. However, as pointed out by Gallagher (2001) in IT, they are not only primary in a developmental sense, but also as an experiential second-person perspective of interaction.

The case study has shown that an intuitive form of understanding emerges when interacting with the other, from sharing an internal state of engagement (as described by Reddy, 2010), which results from the reciprocity or complementarity of action. We have proposed a description of this process based on free-energy principle (FEP) theory. That is, the baby's sense of the mother's intention would be accounted for by how much surprised (in a FEP theory formulation sense) the baby is feeling while enacting in the dyad. Hence, the less surprised the baby feels, the more he/she would be in tune with the mother's intention. So understanding the mother's intention would constitute a process informed by an emergent internal state that the baby is able to experience on-line when interacting with her. Consequently, from our interpretation of the concept of *active inference*, understanding is viewed as a process of coupling, in which neither agent resorts to reading the “inner” or “mental” goals, beliefs, and desires of the other. Instead, understanding the other, in the form of emerging coordination, is achieved through direct perception of embodied activity.

This way of understanding is not guaranteed to be successful all the time, as well as for all sort of subjects/robots. When analysing Figure 7, it is possible to notice that occasionally the human and the robot intentions were easily synchronized (e.g., in the events 1, 6, and 7 their intentions became similar early in the interaction interval). Other times, the human persisted for a while before human-to-robot alternations could take place (e.g., in the events 2, 3, and 5 the robot changed its intention according to the human's intentions). Finally, at times the human had to adapt to the robot's intentions, so robot-to-human alternations were produced (e.g., the events 4 and 8). Therefore, the duration required for a change in either side would depend on the characteristics of both partners and the dynamics of interaction. If the agent has a weaker belief, its adaptation becomes faster. On the other hand, if the agent has a stronger belief, its adaptation becomes slower (Chame and Tani, 2020; Ohata and Tani, 2020).

These results present some similarities with respect to situations described in mother-infant interactions (Trevarthen, 1979). They suggest that in our study the robot is occasionally able to exhibit strong intentionality (or behave subjectively), whereas in other situations it adapts to the subjectivity of the human counterpart by modifying its own intentionality (they demonstrate the capacity of intersubjectivity). We believe that through the design of human robot experiments of this sort, it is possible to study diverse aspects of social cognition, such as the emergence of consensus in intuitive non-verbal communication. As it was explained previously, the robot was not trained to learn how to shift from the generation of one behavior to another. Such changes resulted from the human actions on its body, and from its capacity of perceiving action affordances that make sense in the context of interaction.

## 5. Discussion: Perspectives for Human Science

The previous sections have presented the foundations and the proposal of a methodological resource for studying human-robot interaction, through the modeling of neural cognitive control, from artificial recurrent neural networks. An experiment was described where a human subject interacted with a virtual Cartesian robot via the computer mouse, in order to illustrate the potentialities of our approach. In this section, we argue on possible implications and perspectives for human science research. Notably, we focus on the fields of developmental psychology, education technology, and cognitive rehabilitation.

### 5.1. Developmental Psychology

The introduction section has contextualized our research interests in the domain of enactivist social cognition. Notably, in the study of *primary intersubjectivity*. Thus, we have been inspired by Trevarthen's influential work where maternal-infant interactions were recorded and analyzed (Trevarthen, 1979). The work constituted a fundamental criticism to theory of mind accounts of social cognition. It described intuitive communication in dyadic interactions as a shared control situation, where individuals reciprocally influence each other.

Although interaction theories of social cognition are not consensual, there is a shared interest in studying dyadic interaction as a second person perspective level of experience, which corresponds to forms of mutual relatedness, co-experiencing, or intersubjective experiencing through reciprocal interaction. Our research is consistent with such agenda. Hence, as shown in the case study experiment, the considered scenario is characterized by direct interaction, where the human modified the body posture of the virtual robot and received visual feedback on the robot's motion. Hence, the human tried to communicate with the robot through corporal patterns or gestures. We have shown in related studies with real robots (Chame and Tani, 2020) that this relation can be studied both ways in the physical dimension, so the human and the robot are able to modify each other's body posture.

We have described interaction enactment as the agent's capability of taking deliberative action while conforming or adjusting to the actions induced by the partner. Hence, behavior emerges only partly under the volitional control of the agent. We have proposed to study such dynamics from an interpretation of FEP theory which is consistent with autopoietic enactivist social cognition. Consequently, we investigate perception, cognition, and action as explained by an hermeneutic circle in dyadic encounters.

Given the context where IT emerged (i.e., opposing a cognitivist view of social cognition), assumptions based on knowledge representation and inference have been criticized when studying primary intersubjectivity. This is pointed out by Reddy (2018) when analyzing assumptions about the nature and availability of mind that views early human communication as a process where the infant infers other's hidden mental states. Grounded in FEP theory, our work does not study primary intersubjectivity as a theory of mind skill. We have argued that internal representation and prediction are considered in the generative sense, from the organismic autopoietic self-organization. As pointed out by Bruineberg and Rietveld (2019), perception and action from anticipatory dynamics and free-energy minimization ensure the agent with the capability of maintaining adaptive sensibility to relevant affordances in a given context, which would not be about reconstructing the structural hidden causes of the other's mind. Hence, the agent perceives action affordances characterized by intention, and tries to fulfill them to make sense in the interaction by taking motor action.

Perhaps an interesting direction of research would be studying the developmental aspect of human primary intersubjectivity skills in human-robot interaction with subjective robots. As pointed out by Torres et al. (2013), studying interaction is certainly subject to considerable methodological difficulties, so behavior measures of physical movements are rarely included. We believe that behavior measures could be complemented with the inclusion of the subjective dimension of the robot partner, so achieving more informed psychometric observations on real-time interaction skills. This can be a relevant perspective for developmental psychology, since most psychometric instruments evaluate interaction with static objects. Therefore, intra-subject measurements could be compared, in a longitudinal study sense, to observe the subject's development. Additionally, intersubject comparisons could be constituted based on how subjects individually interact with a particular robotic prototype.

NRL is a framework conceived for constant evolution, and incorporation of novel neural network architectures. Ongoing efforts in our lab are focusing on exploring the modulation and understanding of prior bias or believe underlying behavior. As it is described in the last part of the Appendix B section, the PV-RNN architecture, which was taken as a case study in this work, is currently been extended for investigating initial sensitivity in goal directed behavior.

Through the proposal of NRL, our work with neural agents has described in detail the dynamics of action deliberation. It could be argued that our proposal could have invested some efforts in detailing the dynamics of action compliance. This aspect was left out of scope mainly due to the fact that robotic platforms are diverse. Furthermore, commonly affordable robots are designed under the classical engineering modeling approach, including conventional control schemes and architectures. Since real-time responsiveness is a fundamental aspect, it is somehow problematic proposing a control model that would run over a virtual abstraction of the host native platform. Hopefully, in the near future more bio-inspired robotic structures, capable of evolving in the phylogenetic sense, would be available for most research labs.

A final aspect to be discussed is that developmental psychology studies in mother-infant interaction have reported early behavior patterns as rudimentary, much distinct from the stylized motions proposed in the case study outlining the body shape of a macaw cub, which the artificial neural model learned. Although it is possible to model rudimentary behavior in neural agents, the inclusion of an artistic scenario is perhaps more related to research perspectives on other fields in human science, which is discussed below.

### 5.2. Dynamics, Consensus, and Educational Technology

According to Ackermann (2001), societal convictions on the meanings of being knowledgeable or intelligent, and what it takes to become so, drive attitudes and practices in education. Hence, several theories of learning (e.g., *behaviorism, cognitivism, constructivism, connectivism, constructionism*) have reflected those convictions, and have been central to the contributions of theorists in education (e.g., Bloom, 1956; Siemens, 2005; Freire, 2018).

In education theory, we contextualize our work within the view of *constructionism*, which consists in a philosophy of learning through building artifacts in the world that reflect one's ideas. Undoubtedly, a key influential initiative in approaching technology to education took place in the late sixties, and consisted in the invention of the LOGO programming language in the Massachusetts Institute of Technology (Seymour, 1980). The idea was to include computers in education processes, so that children learn to communicate with computers through a mathematical-logical language, in order to build their own tools and mediations to support their interest within a given context.

*Constructionism* has transcended the virtual world to ground the prototyping of physical agents through the Lego Mindstorm technology (Martin et al., 2000). This platform has been extensively used in the international educational robotics competition *RoboCup*. Notably, in the Junior league^5^, which is dedicated to young scientists mostly enrolled in secondary high-school. The Mindstorm technology has stimulated an explosion of other proposals in assemblage robotics kits.

Interesting questions are raised once new technologies are incorporated into education, in particular, inquiring the actual benefits those technologies provide to distinct academic outcomes. Thus, the aspect of how educational robotics generalizes to areas which are not closely related to the field of robotics itself has been reviewed by Benitti (2012). The review found that most of the studies (around 80%) explored physics, logic, and mathematics related topics, which may suggest a low capacity of absorption to other fields. Moreover, in the light of the increasing availability of machine learning technology (e.g., software libraries, and computational gadgets), one might question the extent to which the complexity of such technology is actually realized by the children. So in practice sophisticated automation prototypes are not simply the result of putting together a set of components which are not really understood by the student (i.e., at the modest cognitive cost of connecting black-box modules).

Our research tries to face the above criticism inspired by efforts in describing the development of cognition and action from a dynamic system theory perspective (e.g., in Thelen and Smith, 1994). Hence, by focusing not only in observing how a given behavior is manifested, but on describing it as a pattern of change over time, and how such change can result from the interaction of multiple subsystems within the individual, the task, and the environment; we hope to be contributing to a reflection on phenomenon which transcends a linear logical causality understanding, to an hermeneutical description of causality that takes place with the self immersion in a feedback loop, so bringing to the foreground the notions of time, intention, stochasticity, and interaction, to a regularly perceived static world.

Perhaps the view of a non-interactive world is still deeply rooted in our society. Arts has provided us with a criticism on the static world view, though forms of expressions such as the *abstract expressionist* movement. Hence, the painting technique known as *action painting* (Rosenberg, 1952) reflects the physical act of painting itself, so the work is more the unfolding of an event than a picture. Here, artists employ the forces and momentum generated by their body to paint (an influential exponent in this current is Jackson Pollock, some of his works are *Mural* and *Lucifer*).

Returning to *constructionism*, we believe the modeling of subjective robots, with which communication takes place through negotiation in shared behavior control, would enrich and extend the interaction scenario envisaged by Papert's seminal ideas. Hence, by keeping in mind the principles of *roboethics* (Tzafestas, 2016), robots could be conceived as systems capable of intention. We hope this sort of synthetic agent would become relevant to other fields of knowledge beyond educational technology. An example is rehabilitation learning, which is discussed next.

### 5.3. Cognitive Rehabilitation and Motivation

According to Sohlberg and Mateer (2017), the term *cognitive rehabilitation* follows short when focusing on the aspect of remediation or compensation for decreased cognitive abilities, so the term *rehabilitation of individuals with cognitive impairment* would emphasize more precisely injured individuals (i.e., acquired brain injury, and traumatic brain injury) that are and will continue to be the target of cognitive rehabilitation. In this sense, although a fundamental goal for treatment is improving and compensating cognitive abilities, a larger scope including consequences for the personal, emotional, motivational, and social dimensions of the brain injury, has been incorporated into treatment plans.

Several evidence-based reviews have been conducted with post-stroke cognitive rehabilitation treatment for specific cognitive impairments, and concluded that although there are some evidence in support, the effectiveness of treatments has yet to be established (e.g., memory deficits in das Nair et al., 2016, executive dysfunction in Chung et al., 2013, and attention deficits in Loetscher et al., 2019). Recently, a work by Maier et al. (2020) has proposed virtual reality as a methodology for designing a rehabilitation program in several cognitive domains conjointly, as an alternative to treating cognitive domains in isolation. Along this line, we believe an interesting perspective to follow consists in exploring rehabilitation tasks based on shared control. Our results with VCBot have illustrated the methodological possibility of such integration.

Regarding real robots, the study of Gassert and Dietz (2018), proposes a classification for robotic rehabilitation platforms into: *grounded exoskeletons, grounded end-effector devices*, and *wearable exoskeletons*. These devices are torque-controlled which allows designing diverse interaction tasks involving passive, active-assisted, and active-resisted movements, depending on the treatment goals and the patient's level of impairment. As discussed in Chame and Tani (2020), when considering simultaneous deliberation and adjustment, it is also possible to profile interaction styles from the robot's capacity of taking purposeful actions, while accommodating to the human's intentions. These characteristics could enrich the availability of task repertories. In this way, artificial neuro cognitive control could become an interesting resource for rehabilitation treatments.

A study by Goršič et al. (2017) has explored the benefits of treatments based on interaction, and suggested that playing competitive games with a non-impaired partner has the potential of leading to functional improvement, when compared to conventional exercising, through an increase in motivation and exercising intensity. The study found a less pronounced effect in cooperative games but a positive effect on motivation. Although it is likely that the presence of a human partner plays a role in motivation, in the study it is reported that some subjects preferred to exercise alone. Perhaps a promising line of research would be exploring whether the fact of intersubjective interaction with a robotic partner would lead to higher life quality of subjects, notably, for participants that opted for not interacting with human partners.

Diverse additional studies could be discussed for analysing possible ways neurorobots, capable of active inference, could play a role in rehabilitation. By linking this section with the previous one, we would like to argue the relevance of *constructionism* as potentially contributing to intrinsically motivated engagement of patients in planning, designing tools, and selecting goals. The treatment could also benefit from observing patient's motivation dynamics (e.g., Chame et al., 2019), so a conjoint planning of the treatment between the patient and the therapist is done.

## 6. Conclusions

This work started from the interest in exploring possible ways neurorobots can contribute to advancing the state of the art in human science. For this, our research was contextualized within the field of enactivist social cognition, notably, in the study of control sharing in dyadic interaction, taking place in primary intersubjective communication. We proposed a methodological tool for prototyping robotics agents, modeled from free energy principle theory. Through the proposal of a demonstration program for interacting, we have shown the potentialities of our methodology for real-time human-robot interaction experiments. Finally, we discussed three main perspectives for human science. Firstly, we have argued for the inclusion of neurorobotics as a resource for investigating embodied social cognition in developmental psychology. Secondly, we have discussed how our proposal is related to the theory of constructionism in education, by contributing to move from the learning of linear logical causality, to a circular understanding of causality that takes place in the subject immersion in a feedback loop when building and interacting with neurorobots. Finally, we have argued on the relevance of neurorobots for the field of cognitive rehabilitation, and how shared control interactive tasks could complement treatment methodologies in physical and virtual environments.

## Ethics Statement

Ethical review and approval was not required for the study on human participants in accordance with the local legislation and institutional requirements. Written informed consent for participation was not required for this study in accordance with the national legislation and the institutional requirements.

## Author Contributions

HC and JT proposed the theoretical conceptualization of the project within the study of primary intersubjectivity. HC designed the NRL computational architecture and the VCBot client program, implemented the projects, and conducted the study case evaluation. AA and JT proposed the PV-RNN framework and provided insightful tips on the mathematical modeling, experimentation, and performance. JT supervised the project. All authors contributed to the article and approved the submitted version.

## Conflict of Interest

The authors declare that the research was conducted in the absence of any commercial or financial relationships that could be construed as a potential conflict of interest.

## Footnotes

^1^Understood as the persistence of self-organization of an organism given its characteristic dynamical structure, while interacting with the environment.

^2^*Ergodicity* implies that the average probability of a system being in a given state is equivalent to the probability of being in such state when observed randomly.

^3^By considering computational requirements from previous experiences with diverse robot tasks (e.g., manipulation in Chame and Martinet (2015), human inspired locomotion and object approach in Chame and Martinet (2016a), walk with top-down and bottom up visual attention in Chame and Martinet (2016b), the C++ programming language (Stroustrup, 2020) was selected for developing the NRL project. The software is released under the terms of the 3-Clause BSD License in the github repository https://github.com/oist-cnru/NRL.

^4^The program is released under the terms of the 3-Clause BSD License at the github repository https://github.com/oist-cnru/VCBot. VCBot is modeled as a two degrees of freedom robot articulated by prismatic joints (Khalil and Dombre, 2002). The software is implemented as a client application in the python programming language (Van Rossum and Drake, 2011), which runs on the top of the NRL back-end. The project is documented in details, with descriptions on the operation modes available. Tutorial videos for installation and operation are proposed in the project's repository.

^5^https://www.robocup.org
