# Supplementary material for: A Hybrid Human-Neurorobotics Approach to Primary Intersubjectivity via Active Inference
Source: Front Psychol. 2020 Dec 1;11:584869. doi: 10.3389/fpsyg.2020.584869 (PMC7736637; doi:10.3389/fpsyg.2020.584869)
Supplement: Supplementary file 1 [file Data_Sheet_1.pdf]

# APPENDIXES

## A: The neural robotics library (NRL)

The *neural robotics library* (NRL) is a project designed to serve as an open-source tool for interdisciplinary research in human social cognition. NRL is structured according to the four-step methodology proposed in Figure 1. The project focuses on providing neural robots with the capability of deliberation control for interaction (see Figure *Interaction dynamics* in the article's section 3). The software is released at the github repository[1] under the terms of the 3-Clause BSD License. NRL considered recommendations for systems and software engineering developments by the standard ISO/IEC 25010 (Iso (2011)).

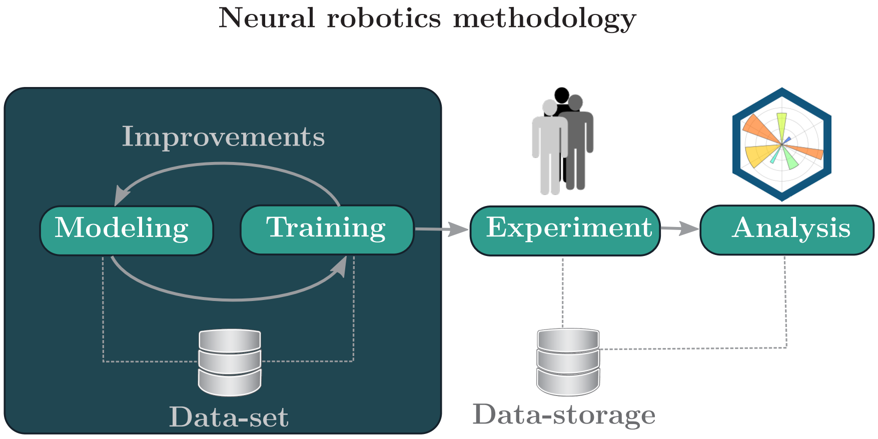


**Figure 1.** The researcher develops a prototype of the neural agent by setting the model's parameters, collecting the behavior dataset, and training the model. In the experimental phase, data from interaction with the subjects is registered, and lately analyzed with the help of graphical and statistical tools.

Prototyping robotic experiments is a challenging endeavor. Provided that robots actuate on the environment, it is fundamental to consider safety, for this, run-time errors must be carefully anticipated and handled. Human-robot interaction presents the supplementary difficulty of requiring computation efficiency for real-time performance. By considering previous experiences with diverse robot tasks (e.g. manipulation in Chame and Martinet (2015), human inspired locomotion and object approach in Chame and Chevallereau (2016a), walk with top-down and bottom up visual attention in Chame and Chevallereau (2016b)), the C++ programming language (Stroustrup (2000)) was selected for developing the NRL project.

In terms of abstraction from the hardware platform resources, C++ is considered an intermediate level language, which is adequate for situations where the programmer intends to control how low level machine resources are used, such as how memory is allocated and information is retrieved. Conveniently, the language syntax conforms to the Object Oriented software engineering paradigm (Meyer (1997)), which favors the quality of software maintainability.

Figure 2 presents the Object Oriented software design. As noticed, in NRL network and layer behaviors are abstracted, respectively, in the INetwork and the ILayer interfaces. In this manner, new functionalities can be added without interfering significantly with available implementations.

[1] Project repository: https://github.com/oist-cnru/NRL

The network and layers exchange information through the IContext interface. Extended classes would act as data buffers, so information can be shared among other levels of the layered hierarchy. An extended description of the software design is provided in the project's repository.

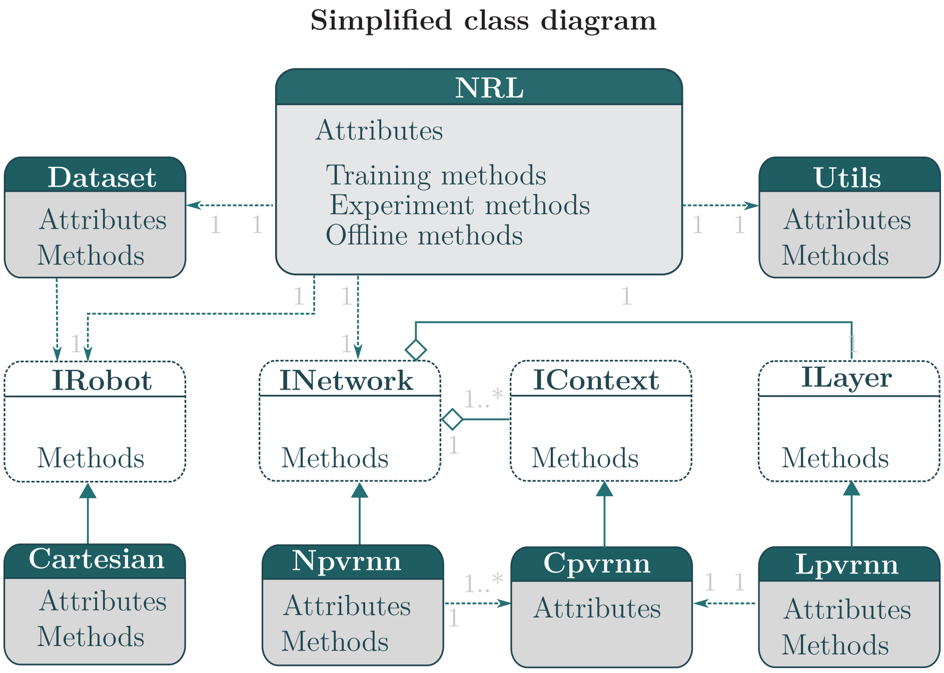


**Figure 2.** Abstract classes are shown in white background.

In order to ensure compatibility, portability, and reducing the chances of run-time errors, NRL relies on free software platforms developed and maintained independently by third parties. Such resources are largely used in the scientific domain. Hence, since the mathematical modeling of artificial neural networks involves a fair amount of linear algebra, the project considered the Eigen template headers for linear algebra version 3.3 developed at INRIA by Guennebaud and Jacob (2013). Also, NRL includes the C++ Standard Library, which are available under multiple platforms.

NRL is a general framework that can be extended to include diverse recurrent neural network architectures. In this work the network type PV-RNN is taken as a case study. The reader interested in consulting the mathematical formalism of PV-RNN is referred to Ahmadi and Tani (2019). For self-containment, the next Appendix section summarizes the mathematical model. The library has been used in real experiments, as reported in the study by Chame and Tani (2020), which included the humanoid Torobo.

## B: The PV-RNN framework

The following sections provide the mathematical details of PV-RNN architectures. The reader is invited to consult the work by Ahmadi and Tani (2019) for more details. In PV-RNN two information processes are involved. The generative process follows a top-down information flow. It is in charge of anticipating the sensory state $\boldsymbol{x}_t$ at time $t$ from prior hidden latent representations $P_\phi$ within the network context $c_t$. On the other side, the inference process involves latent posterior distributions $Q_\pi$. It consists in a bottom-up computation flow, where the surprise signal is back propagated

through time (BPTT) in the network hierarchy, within a sliding temporal window $s_t$. Algorithm 1 describes how deliberation control is achieved in the neural robot.

**Algorithm 1** Deliberation control

```
procedure doInteraction
    t ← 0
    c_t, x_t, s_t ← initialize()
    while t ≤ T^experiment do
        t ← t + 1
        x_t ← doGeneration(P_φ, c_t)
        doMotorControl(x_t)
        if t > S^size then
            s_t ← [s_{2:S^size}, x_t]
            c_{t+1} ← doInference(Q_π, s_t, c_{t−S^size}, S^size)
        else
            s_t ← [s_{1:t}, x_t]

procedure doInference(Q_π, s^y, c, n)
    ELBO ← initialize()
    for e ← 1, N^epochs do
        s^x, s^c ← doGeneration(Q_π, c, n)
        ELBO ← doBPTT(s^x, s^y, s^c)
    ← s_t^c
```

## The Generative model

Let[2] the generative model $P_\phi$ be defined from the parameters $\phi$, distributed among the components: generated prediction $\boldsymbol{x}$, stochastic $\boldsymbol{z}$ and the deterministic $\boldsymbol{d}$ latent states. Given the generative model of PV-RNN (see Fig. 3), for a prediction $\boldsymbol{x}_{1:T} = (\boldsymbol{x}_1, \boldsymbol{x}_2, ..., \boldsymbol{x}_T)$, and considering the parameters $\phi_{\mathrm{x}}$, $\phi_{\mathrm{z}}$, and $\phi_{\mathrm{d}}$, $P_\phi$ factorizes such that:

$$P_\phi\left(\boldsymbol{x}_{1:T}, \boldsymbol{z}_{1:T}, \boldsymbol{d}_{1:T} | \boldsymbol{z}_0, \boldsymbol{d}_0\right) = \prod_{t=1}^{T} P_{\phi_{\mathrm{x}}}\left(\boldsymbol{x}_t | \boldsymbol{d}_t\right) P_{\phi_{\mathrm{z}}}\left(\boldsymbol{z}_t | \boldsymbol{d}_{t-1}\right) P_{\phi_{\mathrm{d}}}\left(\boldsymbol{d}_t | \boldsymbol{d}_{t-1}, \boldsymbol{z}_t\right) \tag{1}$$

Let the deterministic states be defined according to a MTRNN structure (Yamashita and Tani (2008)). For the $k^{\mathrm{th}}$ context layer at time $t$, with timescale $\iota^k$, the internal dynamics are represented such that

$$\boldsymbol{h}_t^k = \left(1 - \frac{1}{\iota^k}\right) \boldsymbol{h}_{t-1}^k + \frac{1}{\iota^k}\left(\boldsymbol{W}_{\mathrm{dh}}^{kk} \boldsymbol{d}_{t-1}^k + \boldsymbol{W}_{\mathrm{dh}}^{kk-1} \boldsymbol{d}_{t-1}^{k-1} + \boldsymbol{W}_{\mathrm{dh}}^{kk+1} \boldsymbol{d}_{t-1}^{k+1} + \boldsymbol{W}_{\mathrm{zh}}^{kk} \boldsymbol{z}_t^k + \boldsymbol{b}_{\mathrm{h}}^k\right), \tag{2}$$

[2] Notation: layer's latent states are denoted bold low-case, biases are denoted $\boldsymbol{b}$, weight connections are denoted $\boldsymbol{W}$ with subscripts indicating the origin and destination of the connection (e.g., $\boldsymbol{W}_{\mathrm{zd}}$ are the weights connecting z to d units). Superscripts $k \in \{1, ..., K\}$ indicate from low to high, the layer's level in the MTRNN hierarchy. Finally, the superscripts p and q are used to distinguish between variables that belong to the prior and posterior distributions, respectively.

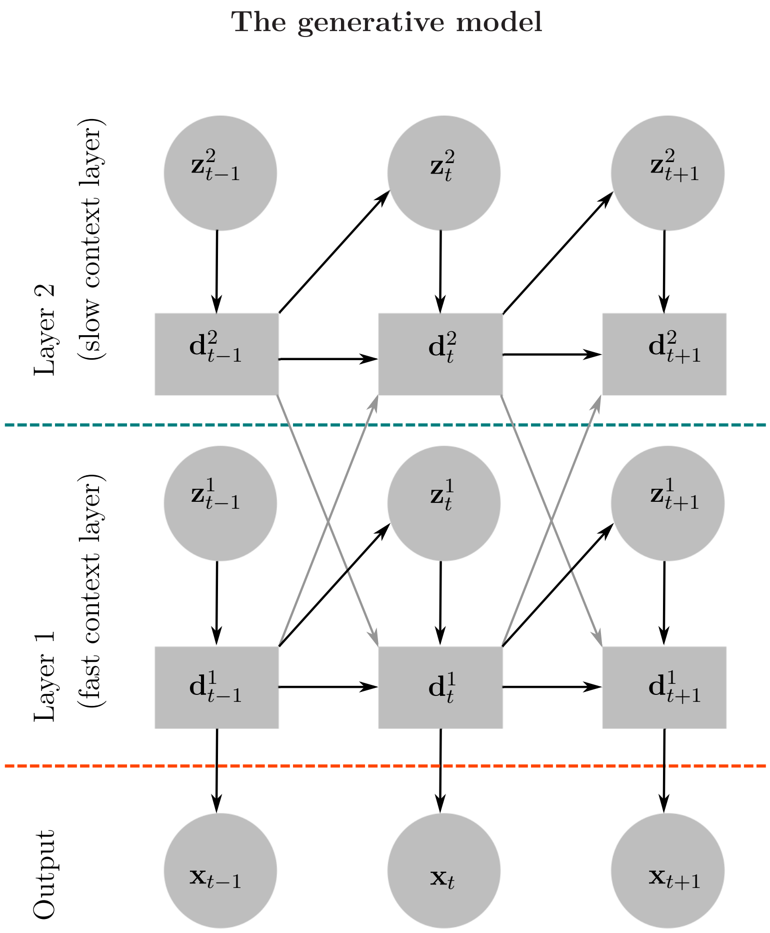


**Figure 3.** Graph representation for the generative model of the PV-RNN framework (Ahmadi and Tani (2019)) in a MTRNN setting (Yamashita and Tani (2008)). A two-level layer hierarchy is shown for illustration.

$$\boldsymbol{d}_t^k = \tanh\left(\boldsymbol{h}_t^k\right). \tag{3}$$

The prior distribution $P_{\phi_z}(\boldsymbol{z}_t|\boldsymbol{d}_{t-1})$ is modeled as a Gaussian with diagonal covariance matrix, such that

$$P_{\phi_z}(\boldsymbol{z}_t|\boldsymbol{d}_{t-1}) = \mathcal{N}\left(\boldsymbol{z}_t; \boldsymbol{\mu}_t^{\mathrm{p}}, \boldsymbol{\sigma}_t^{\mathrm{p}}\right), \tag{4}$$

where $\boldsymbol{\mu}_t^{\mathrm{p}}$ and $\boldsymbol{\sigma}_t^{\mathrm{p}}$ are, respectively, the mean and standard deviation of $\boldsymbol{z_t} = \boldsymbol{\mu}_t^{\mathrm{p}} + \boldsymbol{\sigma}_t^{\mathrm{p}} * \boldsymbol{\epsilon}$, with $\boldsymbol{\epsilon}$ sampled from $\mathcal{N}(0,1)$. The variables $[\boldsymbol{\mu}_t^{\mathrm{p}}, \log(\boldsymbol{\sigma}_t^{\mathrm{p}})] = f_{\phi_z}(\boldsymbol{d}_{t-1})$ are obtained with $f_{\phi_z}(.)$ the one layer feed-forward neural network, such that

$$\boldsymbol{u}_t^{\mathrm{p},k} = \boldsymbol{W}_{\mathrm{d}\mu}^{\mathrm{p},kk} \boldsymbol{d}_{t-1}^k + \boldsymbol{b}_\mu^{\mathrm{p},k}, \tag{5}$$

$$\boldsymbol{\mu}_t^{\mathrm{p},k} = \tanh\left(\boldsymbol{u}_t^{\mathrm{p},k}\right), \tag{6}$$

$$\log\left(\boldsymbol{\sigma}_t^{\mathrm{p},k}\right) = \boldsymbol{W}_{\mathrm{d}\sigma}^{\mathrm{p},kk}\boldsymbol{d}_{t-1}^{k} + \boldsymbol{b}_{\sigma}^{\mathrm{p},k}. \tag{7}$$

### The inference model

Let the inference model $Q_\pi$ (the approximate posterior) be defined from the parameters $\pi$, such that

$$Q_\pi(\boldsymbol{z}_t|\boldsymbol{d}_{t-1}, \boldsymbol{e}_{t:T}) = \mathcal{N}\left(\boldsymbol{z}_t; \boldsymbol{\mu}_t^{\mathrm{q}}, \boldsymbol{\sigma}_t^{\mathrm{q}}\right), \tag{8}$$

where $\boldsymbol{\mu}_t^{\mathrm{q}}$ and $\boldsymbol{\sigma}_t^{\mathrm{q}}$ are, respectively, the mean and standard deviation of $\boldsymbol{z_t} = \boldsymbol{\mu}_t^{\mathrm{q}} + \boldsymbol{\sigma}_t^{\mathrm{q}} * \boldsymbol{\epsilon}$, with $\boldsymbol{\epsilon}$ sampled from $\mathcal{N}(0,1)$. The variables $[\boldsymbol{\mu}_t^{\mathrm{q}}, \log\,(\boldsymbol{\sigma}_t^{\mathrm{q}})] = f_{\pi_z}(\boldsymbol{d}_{t-1}, \boldsymbol{a}^{\bar{\mathrm{x}}})$ are obtained with $f_{\pi_z}(.)$ the one layer feed-forward neural network, such that

$$\boldsymbol{u}_t^{\mathrm{q},k} = \boldsymbol{W}_{\mathrm{d}\mu}^{\mathrm{q},kk}\boldsymbol{d}_{t-1}^{k} + \boldsymbol{a}_{\mu,t}^{\bar{\mathrm{x}},k} + \boldsymbol{b}_{\mu}^{\mathrm{q},k}, \tag{9}$$

$$\boldsymbol{\mu}_t^{\mathrm{q},k} = \tanh\left(\boldsymbol{u}_t^{\mathrm{q},k}\right), \tag{10}$$

$$\log\left(\boldsymbol{\sigma}_t^{\mathrm{q},k}\right) = \boldsymbol{W}_{\mathrm{d}\sigma}^{\mathrm{q},kk}\boldsymbol{d}_{t-1}^{k} + \boldsymbol{a}_{\sigma,t}^{\bar{\mathrm{x}},k} + \boldsymbol{b}_{\sigma}^{\mathrm{q},k}. \tag{11}$$

The parameters $\boldsymbol{a}_{1:T}^{\bar{\mathrm{x}},\mathrm{k}}$ are introduced to provide the network with information about the prediction error in relation to a given pattern $\bar{\boldsymbol{x}}$. Thus, $\boldsymbol{a}_{1:T}^{\bar{\mathrm{x}},\mathrm{k}}$ is changed back propagating through time the prediction error $\boldsymbol{e}_{t:T}$, so information about the future steps of $\bar{\boldsymbol{x}}_{t:T}$, and existing dependencies with the current time step $t$, are captured such that

$$\boldsymbol{a}_{\mu,t}^{\bar{\mathrm{x}},k} = \boldsymbol{a}_{\mu,t}^{\bar{\mathrm{x}},k} + \alpha\frac{\partial L}{\partial \boldsymbol{a}_{\mu,t}^{\bar{\mathrm{x}},k}}, \tag{12}$$

$$\boldsymbol{a}_{\sigma,t}^{\bar{\mathrm{x}},k} = \boldsymbol{a}_{\sigma,t}^{\bar{\mathrm{x}},k} + \alpha\frac{\partial L}{\partial \boldsymbol{a}_{\sigma,t}^{\bar{\mathrm{x}},k}}, \tag{13}$$

with $\alpha$ denoting the learning rate.

Let the Variational Evidence Lower Bound (ELBO) $L(\phi,\pi)$ be defined by

$$L(\phi,\pi) = \sum_{t=1}^{T}\left(\frac{1}{n_{\mathrm{x}}}\underbrace{E_{\mathrm{Q}_\pi}\left[\log P_{\phi_x}\left(\boldsymbol{x}_t|\tilde{\boldsymbol{d}}_t, \boldsymbol{z}_t\right)\right]}_{\text{accuracy/reconstruction error}} - \frac{w}{n_{\mathrm{z}}}\underbrace{\mathrm{KL}\left[Q_\pi\left(\boldsymbol{z}_t|\tilde{\boldsymbol{d}}_{t-1}, \boldsymbol{e}_{t:T}\right)\|P_{\phi_z}\left(\boldsymbol{z}_t|\tilde{\boldsymbol{d}}_{t-1}\right)\right]}_{\text{complexity/regulation error}}\right). \tag{14}$$

Maximizing the ELBO is mathematically equivalent to minimizing free energy. Here $n_{\mathrm{x}}$ is the number of degrees of freedom of the robot, and $n_{\mathrm{z}}$ is the total number of z units considering all layers. Since $\boldsymbol{d}_t$ is deterministic given $\boldsymbol{d}_{t-1}$ and $\boldsymbol{z}_t$, $\tilde{\boldsymbol{d}}_t$ denotes the center of a Dirac distribution. The first term at

the right of the equation is a reconstruction component, it corresponds to the expected log-likelihood under the posterior distribution $Q_\pi$. The second term is a regulation component, it corresponds to the Kullback-Leibler (KL) divergence between the prior and the posterior distributions of the latent variables. The meta-parameter $w$ adjusts the optimization weight in learning the posterior and the prior distributions. From the assumption of Gaussian prior and posterior distributions, after dropping the random variable notation to improve readability, the KL term can be expressed as

$$\mathrm{KL}\left[Q_\pi \| P_{\phi_z}\right] = \log\left(\frac{\boldsymbol{\sigma}^{\mathrm{p}}}{\boldsymbol{\sigma}^{\mathrm{q}}}\right) + \frac{\left(\boldsymbol{\mu}^{\mathrm{p}} - \boldsymbol{\mu}^{\mathrm{q}}\right)^2 + \left(\boldsymbol{\sigma}^{\mathrm{q}}\right)^2}{2\left(\boldsymbol{\sigma}^{\mathrm{p}}\right)^2} - \frac{1}{2}. \tag{15}$$

Finally, the $i^{\mathrm{th}}$ output dimension of the cognitive control space is obtained from the one layer feed-forward propagation of the $\boldsymbol{d}_{i,t}$ units, such that

$$\boldsymbol{o}_{i,t} = \boldsymbol{W}_{\mathrm{dx}_i}\boldsymbol{d}_t^1 + \boldsymbol{b}_{\mathrm{x}_i}. \tag{16}$$

Unlike in Ahmadi and Tani (2019), in this work it is not included in $\boldsymbol{o}_{i,t}$ connections from the stochastic latent distributions at the Low level, in order to reduce the computational complexity. Hence, the output $\boldsymbol{x}_{i,t}$ is such that

$$\boldsymbol{x}_{i,t} = \mathrm{softmax}\left(\boldsymbol{o}_{i,t}\right). \tag{17}$$

Free energy is optimized for distinct purposes during the phases *training* and *experiment* (see Fig. 1). The agent is trained for behavior acquisition in a supervised manner on the behavior primitives. Data can be obtained analytically from mathematically modeling behavior, or captured from direct interaction (e.g. by kinesthetic teaching). Variational models are better at generalizing when compared to deterministic frameworks, so it is not necessary to collect numerous samples on the desired behavior. During the *modeling* phase, the model's meta-parameters are selected to modulate stochasticity (see Table *PV-RNN parameters selection* in the article's section 4.3), resulting in a higher capability of generalization. Free energy optimization in the training phase involves the off-line modification of parameters including synaptic weights, biases, and state variables. Contrarily, the *experiment* phase is characterized by on-line inference, where only state variables within the temporal sliding window are optimized (see Figure *Interaction dynamics* in the article's section 3). The objective of free-energy optimization during experiment is to allow the agent to perceive action affordances in intentional interaction, through changes in the overall PV-RNN latent state. Next, the model gradients for the BPTT algorithm are provided.

## BPTT gradients

In this section the back propagation through time gradients are provided. The variable change $\boldsymbol{\rho} = \log(\boldsymbol{\sigma})$ is proposed to improve readability. By applying the derivation chain rule, the gradients are computed from Eq. (14), such as:

$$\frac{\partial \boldsymbol{L}_{i,t}}{\partial \boldsymbol{o}_{i,t}} = \frac{1}{n_{\mathrm{x}}}\left(\boldsymbol{x}_{i,t} - \bar{\boldsymbol{x}}_{i,t}\right), \tag{18}$$

$$\frac{\partial \boldsymbol{L}_t}{\partial \boldsymbol{d}^k_{i,t-1}} = \sum_s \frac{\partial \boldsymbol{L}_t}{\partial \boldsymbol{o}_{s,t-1}} \frac{\partial \boldsymbol{o}_{s,t-1}}{\partial \boldsymbol{d}^k_{i,t-1}} + \sum_j \frac{\partial \boldsymbol{L}_t}{\partial \boldsymbol{h}^k_{j,t}} \frac{\partial \boldsymbol{h}^k_{j,t}}{\partial \boldsymbol{d}^k_{i,t-1}} + \sum_y \frac{\partial \boldsymbol{L}_t}{\partial \boldsymbol{h}^{k-1}_{y,t}} \frac{\partial \boldsymbol{h}^{k-1}_{y,t}}{\partial \boldsymbol{d}^k_{i,t-1}} + \sum_r \frac{\partial \boldsymbol{L}_{i,t}}{\partial \boldsymbol{h}^{k+1}_{r,t}} \frac{\partial \boldsymbol{h}^{k+1}_{r,t}}{\partial \boldsymbol{d}^k_{i,t-1}} + \sum_m \frac{\partial \boldsymbol{L}_t}{\partial \boldsymbol{\mu}^{\mathrm{p},k}_{m,t}} \frac{\partial \boldsymbol{\mu}^{\mathrm{p},k}_{m,t}}{\partial \boldsymbol{d}^k_{i,t-1}} + \sum_m \frac{\partial \boldsymbol{L}_t}{\partial \boldsymbol{\rho}^{\mathrm{p},k}_{m,t}} \frac{\partial \boldsymbol{\rho}^{\mathrm{p},k}_{m,t}}{\partial \boldsymbol{d}^k_{i,t-1}} + . \sum_m \frac{\partial \boldsymbol{L}_t}{\partial \boldsymbol{\mu}^{\mathrm{q},k}_{m,t}} \frac{\partial \boldsymbol{\mu}^{\mathrm{q},k}_{m,t}}{\partial \boldsymbol{d}^k_{i,t-1}} + \sum_m \frac{\partial \boldsymbol{L}_t}{\partial \boldsymbol{\rho}^{\mathrm{q},k}_{m,t}} \frac{\partial \boldsymbol{\rho}^{\mathrm{q},k}_{m,t}}{\partial \boldsymbol{d}^k_{i,t-1}} \tag{19}$$

The terms involving other levels of the hierarchy are set to zero when not defined (i.e. in the case of level $k+1$ in the highest layer or the level $k-1$ in the lowest layer). Likewise, the gradient below is only defined for $k=1$

$$\frac{\partial \boldsymbol{o}_{s,t-1}}{\partial \boldsymbol{d}^k_{i,t-1}} = \boldsymbol{W}^{kk}_{\mathrm{dx}_{s,i}}, \tag{20}$$

$$\frac{\partial \boldsymbol{L}_t}{\partial \boldsymbol{h}^k_{i,t-1}} = \frac{\partial \boldsymbol{L}_t}{\partial \boldsymbol{d}^k_{i,t-1}} \frac{\partial \boldsymbol{d}^k_{i,t-1}}{\partial \boldsymbol{h}^k_{i,t-1}} + \frac{\partial \boldsymbol{L}_t}{\partial \boldsymbol{h}^k_{i,t}} \frac{\partial \boldsymbol{h}^k_{i,t}}{\partial \boldsymbol{h}^k_{i,t-1}}, \tag{21}$$

$$\frac{\partial \boldsymbol{d}^k_{i,t-1}}{\partial \boldsymbol{h}^k_{i,t-1}} = 1 - \tanh^2(\boldsymbol{h}^k_{i,t}), \tag{22}$$

$$\frac{\partial \boldsymbol{h}^k_{i,t}}{\partial \boldsymbol{h}^k_{i,t-1}} = 1 - \frac{1}{\iota^k}, \tag{23}$$

$$\frac{\partial \boldsymbol{h}^k_{j,t}}{\partial \boldsymbol{d}^k_{i,t-1}} = \frac{1}{\iota^k} \boldsymbol{W}^{kk}_{\mathrm{hd}_{j,i}}, \tag{24}$$

$$\frac{\partial \boldsymbol{h}^{k-1}_{y,t}}{\partial \boldsymbol{d}^k_{i,t-1}} = \frac{1}{\iota^{k-1}} \boldsymbol{W}^{kk-1}_{\mathrm{dh}_{y,i}}, \tag{25}$$

$$\frac{\partial \boldsymbol{h}^{k+1}_{r,t}}{\partial \boldsymbol{d}^k_{i,t-1}} = \frac{1}{\iota^{k+1}} \boldsymbol{W}^{kk+1}_{\mathrm{dh}_{r,i}}, \tag{26}$$

$$\frac{\partial \boldsymbol{L}_t}{\partial \boldsymbol{\mu}^{\mathrm{p},k}_{m,t}} = \frac{w^k}{n_{\mathrm{z}}} \left( \frac{\boldsymbol{\mu}^{\mathrm{p},k}_{m,t} - \boldsymbol{\mu}^{\mathrm{q},k}_{m,t}}{(\boldsymbol{\sigma}^{\mathrm{p},k}_{m,t})^2} \right), \tag{27}$$

$$\frac{\partial \boldsymbol{\mu}^{\mathrm{p},k}_{m,t}}{\partial \boldsymbol{d}^k_{i,t-1}} = \left(1 - \tanh^2\left(\boldsymbol{u}^{\mathrm{p},k}_{m,t}\right)\right) \boldsymbol{W}^{kk}_{\mu^{\mathrm{p}} d_{m,i}}, \tag{28}$$

$$\frac{\partial \boldsymbol{L}_t}{\partial \boldsymbol{\rho}^{\mathrm{p},k}_{m,t}} = \frac{w^k}{n_{\mathrm{z}}} \left( 1 - \frac{\left( \left( \boldsymbol{\mu}^{\mathrm{q},k}_{m,t} - \boldsymbol{\mu}^{\mathrm{p},k}_{m,t} \right)^2 + \left( \boldsymbol{\sigma}^{\mathrm{q},k}_{m,t} \right)^2 \right)}{\left( \boldsymbol{\sigma}^{\mathrm{p},k}_{m,t} \right)^2} \right), \tag{29}$$

$$\frac{\partial \boldsymbol{\rho}^{\mathrm{p},k}_{m,t}}{\partial \boldsymbol{d}^{k}_{i,t-1}} = \boldsymbol{W}^{kk}_{\sigma^{\mathrm{p}}\mathrm{d}_{m,i}}, \tag{30}$$

$$\frac{\partial \boldsymbol{L}_t}{\partial \boldsymbol{\mu}^{\mathrm{q},k}_{m,t}} = \frac{\partial \boldsymbol{L}_t}{\partial \boldsymbol{z}^{\mathrm{q},k}_{m,t}} + \frac{1}{n_{\mathrm{z}}} \frac{\boldsymbol{\mu}^{\mathrm{q},k}_{m,t} - \boldsymbol{\mu}^{\mathrm{p},k}_{i,t}}{\left( \sigma^{\mathrm{p},k}_{m,t} \right)^2}, \tag{31}$$

$$\frac{\partial \boldsymbol{L}_t}{\partial \boldsymbol{z}^{\mathrm{q},k}_{m,t}} = \sum_j \frac{\partial \boldsymbol{L}_t}{\partial \boldsymbol{h}^{k}_{m,t}} \left( \frac{1}{\iota^k} \boldsymbol{W}^{kk}_{\mathrm{zh}_{m,i}} \right), \tag{32}$$

$$\frac{\partial \boldsymbol{\mu}^{\mathrm{q},k}_{m,t}}{\partial \boldsymbol{d}^{k}_{i,t-1}} = \left( 1 - \tanh^2 \left( \boldsymbol{u}^{\mathrm{q},k}_{m,t} \right) \right) \boldsymbol{W}^{kk}_{\mu^{\mathrm{q}}d_{m,i}}, \tag{33}$$

$$\frac{\partial \boldsymbol{L}_t}{\partial \boldsymbol{\rho}^{\mathrm{q},k}_{m,t}} = \frac{\partial \boldsymbol{L}_t}{\partial \boldsymbol{z}^{\mathrm{q},k}_{m,t}} \boldsymbol{\epsilon}^{\mathrm{q},k}_{m,t} \boldsymbol{\sigma}^{\mathrm{q},k}_{m,t} + \frac{w^k}{n_{\mathrm{z}}} \left( -1 + \frac{(\boldsymbol{\sigma}^{\mathrm{q},k}_{m,t})^2}{(\boldsymbol{\sigma}^{\mathrm{p},k}_{m,t})^2} \right), \tag{34}$$

$$\frac{\partial \boldsymbol{\rho}^{\mathrm{q},k}_{m,t}}{\partial \boldsymbol{d}^{k}_{i,t-1}} = \boldsymbol{W}^{kk}_{\sigma^{\mathrm{q}}\mathrm{d}_{m,i}}, \tag{35}$$

$$\frac{\partial \boldsymbol{L}_t}{\boldsymbol{a}^{\bar{\mathrm{x}},k}_{\mu_{i,t}}} = \left( 1 - \tanh^2 \left( \boldsymbol{u}^{\mathrm{q},k}_{i,t} \right) \right) \frac{\partial \boldsymbol{L}_t}{\partial \boldsymbol{\mu}^{\mathrm{q},k}_{i,t}}, \tag{36}$$

$$\frac{\partial \boldsymbol{L}_t}{\boldsymbol{a}^{\bar{\mathrm{x}},k}_{\sigma_{i,t}}} = \frac{\partial \boldsymbol{L}_t}{\partial \boldsymbol{\rho}^{\mathrm{q},k}_{i,t}}. \tag{37}$$

The synaptic weights and biases are updated such that

$$\frac{\partial \boldsymbol{L}_t}{\partial \boldsymbol{W}^{kk}_{\mathrm{dh}_{i,j}}} = \sum_t \frac{1}{\iota^k} \boldsymbol{d}^{k}_{j,t-1} \frac{\partial \boldsymbol{L}_t}{\partial \boldsymbol{h}^{k}_{i,t}} \tag{38}$$

$$\frac{\partial \boldsymbol{L}_t}{\partial \boldsymbol{b}^{k}_{\mathrm{h}_i}} = \sum_t \frac{1}{\iota^k} \frac{\partial \boldsymbol{L}_t}{\partial \boldsymbol{h}^{k}_{i,t}} \tag{39}$$

$$\frac{\partial \boldsymbol{L}_t}{\partial \boldsymbol{W}^{kk-1}_{\mathrm{dh}_{i,j}}} = \sum_t \frac{1}{\iota^k} \boldsymbol{d}^{k-1}_{j,t-1} \frac{\partial \boldsymbol{L}_t}{\partial \boldsymbol{h}^{k}_{i,t}} \tag{40}$$

$$\frac{\partial \boldsymbol{L}_t}{\partial \boldsymbol{W}^{kk+1}_{\mathrm{dh}_{i,j}}} = \sum_t \frac{1}{\iota^k} \boldsymbol{d}^{k+1}_{j,t-1} \frac{\partial \boldsymbol{L}_t}{\partial \boldsymbol{h}^{k}_{i,t}} \tag{41}$$

$$\frac{\partial \boldsymbol{L}_t}{\partial \boldsymbol{W}^{\mathrm{p},kk}_{\mathrm{zh}_{i,j}}} = \sum_t \frac{1}{\iota^k} \boldsymbol{z}^k_{j,t} \frac{\partial \boldsymbol{L}_t}{\partial \boldsymbol{h}^k_{i,t}} \tag{42}$$

$$\frac{\partial \boldsymbol{L}_t}{\partial \boldsymbol{W}^{kk}_{\mathrm{d}\mu^{\mathrm{p}}{}_{i,j}}} = \sum_t \left(1 - \tanh^2\left(\boldsymbol{u}^{\mathrm{p},k}_{i,t}\right)\right) \boldsymbol{d}^k_{j,t-1} \frac{\partial \boldsymbol{L}_t}{\partial \boldsymbol{\mu}^{\mathrm{p},k}_{i,t}} \tag{43}$$

$$\frac{\partial \boldsymbol{L}_t}{\partial \boldsymbol{b}^{\mathrm{p},k}_{\mu_i}} = \sum_t \left(1 - \tanh^2\left(\boldsymbol{u}^{\mathrm{p},k}_{i,t}\right)\right) \frac{\partial \boldsymbol{L}_t}{\partial \boldsymbol{\mu}^{\mathrm{p},k}_{i,t}}, \tag{44}$$

$$\frac{\partial \boldsymbol{L}_t}{\partial \boldsymbol{W}^{\mathrm{p},kk}_{\mathrm{d}\sigma^{\mathrm{p}}{}_{i,j}}} = \sum_t \boldsymbol{d}^k_{j,t-1} \frac{\partial \boldsymbol{L}_t}{\partial \boldsymbol{\rho}^{\mathrm{p},k}_{i,t}}, \tag{45}$$

$$\frac{\partial \boldsymbol{L}_t}{\partial \boldsymbol{b}^{\mathrm{p},k}_{\sigma_i}} = \sum_t \frac{\partial \boldsymbol{L}_t}{\partial \boldsymbol{\rho}^{\mathrm{p},k}_{i,t}}. \tag{46}$$

The gradients $\frac{\partial \boldsymbol{L}_t}{\partial \boldsymbol{W}^{\mathrm{p},kk}_{\mathrm{d}\mu_{i,j}}}$, $\frac{\partial \boldsymbol{L}_t}{\partial \boldsymbol{b}^{\mathrm{q},k}_{\mu_i}}$, $\frac{\partial \boldsymbol{L}_t}{\partial \boldsymbol{W}^{\mathrm{q},kk}_{\mathrm{d}\sigma_{i,j}}}$, and $\frac{\partial \boldsymbol{L}_t}{\partial \boldsymbol{b}^{\mathrm{q},k}_{\sigma_i}}$ are computed, analogous to Eqs. (43), (44), (45), and (46).

### PV-RNN extension perspectives

The original formulation of PV-RNN did not consider the possibility of modeling prior believes by the experimenter. An extension under study in our lab consists in dividing the complexity term into a sequence prior part and a unit Gaussian prior part to control the initial sensitivity of the model. Considering the same notation described in Eq. (14), the Variational Evidence Lower Bound (ELBO) $L(\phi, \pi)$ can be defined such that

$$\begin{aligned} L(\phi,\pi) = &\sum_{t=1}^{T} \left(\frac{1}{n_{\mathrm{x}}} E_{\mathrm{Q}_\pi}\left[\log P_{\phi_x}\left(\boldsymbol{x}_t|\tilde{\boldsymbol{d}}_t, \boldsymbol{z}_t\right)\right]\right) - \\ &\sum_{t=2}^{T} \left(\frac{w}{n_{\mathrm{z}}} \mathrm{KL}\left[Q_\pi\left(\boldsymbol{z}_t|\tilde{\boldsymbol{d}}_{t-1}, \boldsymbol{e}_{t:T}\right) \| P_{\phi_z}\left(\boldsymbol{z}_t|\tilde{\boldsymbol{d}}_{t-1}\right)\right]\right) - \frac{w_1}{n_{\mathrm{z}}} \mathrm{KL}\left[Q_\pi\left(\boldsymbol{z}_1|\boldsymbol{e}_1\right) \| P_{\phi_z}\left(\boldsymbol{z}_1\right)\right]. \end{aligned} \tag{47}$$

As noticed, conforming to this new formulation (presented in Ohata and Tani (2020)), along with other changes, two meta-parameters are provided ($w_1$ for time $t = 1$, and $w$ for $t \geq 2$) to adjust the optimization weight in learning the posterior and the prior distributions. This version of PV-RNN has been included in NRL as a network type named *PV-RNN Beta*.

## REFERENCES

Ahmadi, A. and Tani, J. (2019). A novel predictive-coding-inspired variational rnn model for online prediction and recognition. *Neural computation* 31, 2025–2074. doi:10.1162/neco_a_01228

Chame, H. F. and Chevallereau, C. (2016a). Grounding humanoid visually guided walking: From action-independent to action-oriented knowledge. *Information Sciences* 352, 79–97. doi:10.1016/j.ins.2016.02.053

Chame, H. F. and Chevallereau, C. (2016b). A top-down and bottom-up visual attention model for humanoid object approaching and obstacle avoidance. In *2016 XIII Latin American Robotics Symposium and IV Brazilian Robotics Symposium (LARS/SBR)* (IEEE), 25–30

Chame, H. F. and Martinet, P. (2015). Cognitive modeling for automating learning in visually-guided manipulative tasks. In *Informatics in Control, Automation and Robotics* (Springer). 37–53. doi:10.1007/978-3-319-10891-9_2

Chame, H. F. and Tani, J. (2020). Cognitive and motor compliance in intentional human-robot interaction. In *2020 IEEE International Conference on Robotics and Automation (ICRA)* (IEEE), 11291–11297

Guennebaud, G. and Jacob, B. (2013). Eigen: a c++ linear algebra library. *URL http://eigen.tuxfamily. org* Accessed March 3rd 2020

Iso, I. (2011). Iec25010: 2011 systems and software engineering–systems and software quality requirements and evaluation (square)–system and software quality models. *International Organization for Standardization* 34, 2910

Meyer, B. (1997). *Object-oriented software construction*, vol. 2 (Prentice hall New York), second edn.

Ohata, W. and Tani, J. (2020). Investigation of the sense of agency in social cognition, based on frameworks of predictive coding and active inference: A simulation study on multimodal imitative interaction. *Frontiers in Neurorobotics* 14, 61. doi:10.3389/fnbot.2020.00061

Stroustrup, B. (2000). *The C++ programming language* (Pearson Education India)

Yamashita, Y. and Tani, J. (2008). Emergence of functional hierarchy in a multiple timescale neural network model: a humanoid robot experiment. *PLoS computational biology* 4. doi:10.1371/journal.pcbi.1000220
